# Global Analysis of UDP Glucose Pyrophosphorylase (UDPGP) Gene Family in Plants: Conserved Evolution Involved in Cell Death

**DOI:** 10.3389/fpls.2021.681719

**Published:** 2021-06-10

**Authors:** Shuai Liu, Hua Zhong, Qiang Wang, Caixiang Liu, Ting Li, Zhaohua Peng, Yangsheng Li, Hongyu Zhang, Jianglin Liao, Yingjin Huang, Zhaohai Wang

**Affiliations:** ^1^Department of Biochemistry, Molecular Biology, Entomology and Plant Pathology, Mississippi State University, Starkville, MS, United States; ^2^State Key Laboratory of Hybrid Rice, Key Laboratory for Research and Utilization of Heterosis in Indica Rice, Ministry of Agriculture, College of Life Sciences, Wuhan University, Wuhan, China; ^3^Key Laboratory of Crop Physiology, Ecology and Genetic Breeding, Ministry of Education of the People’s Republic of China, Jiangxi Agricultural University, Nanchang, China; ^4^Key Laboratory of Agriculture Responding to Climate Change, Jiangxi Agricultural University, Nanchang, China; ^5^Key Laboratory of Magnetic Resonance in Biological Systems, State Key Laboratory of Magnetic Resonance and Atomic and Molecular Physics, National Centre for Magnetic Resonance in Wuhan, Wuhan Institute of Physics and Mathematics, Innovation Academy of Precision Measurement Science and Technology, Chinese Academy of Sciences, Wuhan, China; ^6^Youth League Committee, Jiangxi Agricultural University, Nanchang, China

**Keywords:** UDP glucose pyrophosphorylase family, UDP-*N*-acetylglucosamine pyrophosphorylase, UDP-glucose pyrophosphorylase, UDP-sugar pyrophosphorylase, conserved evolution, alternative splicing

## Abstract

UDP glucose pyrophosphorylase (UDPGP) family genes have been reported to play essential roles in cell death or individual survival. However, a systematic analysis on UDPGP gene family has not been performed yet. In this study, a total of 454 UDPGP proteins from 76 different species were analyzed. The analyses of the phylogenetic tree and orthogroups divided UDPGPs into three clades, including UDP-*N*-acetylglucosamine pyrophosphorylase (UAP), UDP-glucose pyrophosphorylase (UGP, containing UGP-A and UGP-B), and UDP-sugar pyrophosphorylase (USP). The evolutionary history of the UDPGPs indicated that the members of UAP, USP, and UGP-B were relatively conserved while varied in UGP-A. Homologous sequences of UGP-B and USP were found only in plants. The expression profile of UDPGP genes in *Oryza sativa* was mainly motivated under jasmonic acid (JA), abscisic acid (ABA), cadmium, and cold treatments, indicating that UDPGPs may play an important role in plant development and environment endurance. The key amino acids regulating the activity of UDPGPs were analyzed, and almost all of them were located in the NB-loop, SB-loop, or conserved motifs. Analysis of the natural variants of UDPGPs in rice revealed that only a few missense mutants existed in coding sequences (CDSs), and most of the resulting variations were located in the non-motif sites, indicating the conserved structure and function of UDPGPs in the evolution. Furthermore, alternative splicing may play a key role in regulating the activity of UDPGPs. The spatial structure prediction, enzymatic analysis, and transgenic verification of UAP isoforms illustrated that the loss of N- and C-terminal sequences did not affect the overall 3D structures, but the N- and C-terminal sequences are important for UAP genes to maintain their enzymatic activity. These results revealed a conserved UDPGP gene family and provided valuable information for further deep functional investigation of the UDPGP gene family in plants.

## Introduction

UDP glucose pyrophosphorylase (UDPGP) is a big gene family with three groups, UDP-*N*-acetylglucosamine pyrophosphorylase (UAP), UDP-glucose pyrophosphorylase (UGP), and UDP-sugar pyrophosphorylase (USP). UAP prefers *N*-acetylglucosamine-1-P (GlcNAc-1-P) and *N*-acetylgalactosamine-1-P (GalNAc-1-P) as substrates (Yang et al., 2010; Decker and Kleczkowski, 2019). UGP is reported to be specific for uridine triphosphate (UTP) and glucose 1-phosphate (Glc-1-P) as substrates (Kleczkowski et al., 2010; Decker et al., 2012). For USP, previous studies reported that it had a broader substrate specificity, including galactose-1-phosphate (Gal-1-P), α-glucuronic acid 1-phosphate (GlcA-1-P), and glucose 1-phosphate (Glc-1-P) (Gronwald et al., 2008; Decker and Kleczkowski, 2017). The UDPGPs have been reported related to plant development such as programmed cell death in *Arabidopsis thaliana* (Chivasa et al., 2013), survival in insects (Arakane et al., 2011), and microorganisms (Yi and Huh, 2015), as well as cancers in humans (Itkonen et al., 2015; Wang et al., 2016).

Some studies on UAP were reported to illustrate its function in microorganisms, animals, and plants. UAP was reported in both prokaryotes and eukaryotes, while no homologous sequences were identified between them (Palaka et al., 2019). In fungi, a singular UDP-GlcNAc pyrophosphorylase gene was reported in yeast (*Saccharomyces cerevisiae*), and loss-of-function mutant (*uap1*Δ) exhibited aberrant morphology including swelled or lysed (Mio et al., 1998). In *Moniliophthora perniciosa*, the inhibition of this enzyme leads to cell death (Junior et al., 2013). In insects, the UAP enzyme plays a key role in chitin synthesis (Zhu K.Y. et al., 2016), protein glycosylation (Schimmelpfeng et al., 2006), growth, and development (Zhu K.Y. et al., 2016). Some insects have two members of UAP, and they usually account for different functions. For example, *LdUAP1* regulated the chitin content, while *LdUAP2* managed the development in *Leptinotarsa decemlineata* (Shi et al., 2016). Besides, in the *Locusta migratoria*, the *LmUAP1* inhibited by RNA*i* resulted in mortality, while the *LmUAP2* did not (Liu et al., 2013). In *Drosophila*, the gene *mummy* encodes a UDP-*N*-acetylglucosamine-dipohosphorylase, and the gene mutants exhibited central nervous system fasciculation, dorsal closure, and eye development defects (Schimmelpfeng et al., 2006). Besides, the *mummy* gene also acted as a BMP signaling antagonist (Humphreys et al., 2013). In human, the expression level of *UAP1* is positively correlated with the androgen receptor, which is a main driver of prostate cancer. Inhibition of *UAP1* can specifically sensitize prostate cancer cells to the inhibitors of *N*-linked glycosylation (Itkonen et al., 2015). Few UAP studies were reported in plants. In rice, functional inactivation of *UAP1* was reported to be related to early leaf senescence, defense responses (Wang et al., 2015), and programmed cell death (Xiao et al., 2018).

UGP is a key enzyme in the metabolism of UDP-glucose, which plays an important role in cellulose, callose (Park et al., 2010), sucrose, and polysaccharide synthesis (Meng et al., 2009b). At first, only two homologous genes (*ATUGP1* and *ATUGP2*) were identified belonging to UGP clades in *A. thaliana* with *ATUGP1* predominantly expressed in most tissues (Meng et al., 2008; Meng et al., 2009b). Single mutants (*atugp1* or *atugp2*) did not show any deficiency, while an *atugp1/atugp2* double mutant exhibited extreme deficiency in plant growth and male sterility. Further experiments showed that the destruction of the callose wall around microspores at the tetrad stage gives rise to abnormal development of pollen, resulting in male sterility in the double mutant (Park et al., 2010). In another study, transfer-DNA gene-knockout plants proved that UDP-glucose pyrophosphorylase 1 (*ATUGP1*) regulates fumonisin B1-induced programmed cell death (Chivasa et al., 2013). Then, a novel *UDP-glucose pyrophosphorylase 3* (*AtUGP3*) was reported in *A. thaliana*, which played a key role in sulfolipid biosynthesis (Okazaki et al., 2009). Similarly, two UDP-glucose pyrophosphorylase genes were identified in rice, *UGP1* on chromosome 9 and *UGP2* on chromosome 2 in early research (Chen et al., 2007). Both *UGP1* and *UGP2* were expressed ubiquitously in rice, and the expression level of *UGP1* was much higher than that of *UGP2*. *UGP1* is vital for callose deposition during the stage of pollen mother cells, and when *UGP1* was silenced by RNA interference, the mutant plants exhibited both male sterility and chalky endosperm phenotypic characteristics (Chen et al., 2007). In other plants, such as potato and tobacco, the functions of UGP genes were also reported. Two *UGPs* (*UGP3* and *UGP5*) were reported in potatoes (Katsube et al., 1990; Spychalla et al., 1994), and the studies demonstrated that the *UGP* was associated with cold-sweetening (Sowokinos et al., 2004; Gupta et al., 2008). In tobacco, plant height was significantly increased compared to control lines through overexpressing the UGP gene (Coleman et al., 2006). In *Phaeodactylum tricornutum*, *UGP* was reported to be associated with chrysolaminaran content, lipid biosynthesis, and carbon allocation (Zhu B.H. et al., 2016). In fungi, *UGP* was proven to be associated with oxidative stress response and long-term survival (Yi and Huh, 2015). In *Dictyostelium*, the UDP-glucose derivative plays a key role in autophagic cell death (Tresse et al., 2008). In humans, the loss of function of *UGP2* caused a genetic disease (Roeben et al., 2006), and the *UGP2* expression was correlated with clinicopathological and biological behaviors, which could be used as a biomarker for progression and poor prognosis of gallbladder cancer (Wang et al., 2016). There are a few studies focused on the function of USP. Only one *USP* gene was reported in *Arabidopsis* (*AtUSP*), and a knockout mutant of the *USP* gene exhibited an abnormal development in pollen, resulting in sterility (Schnurr et al., 2006; Geserick and Tenhaken, 2013).

Multiple UGP isoforms have been detected in soybean (Vella and Copeland, 1990), potato (Gupta and Sowokinos, 2003), and rice (Chen et al., 2007). Besides, isoforms of USP were also reported in *Arabidopsis* (Gronwald et al., 2008). However, isoforms of UAP have not been surveyed in plants yet. In the current study, we retrieved and classified UDPGP among plants, animals, and microorganisms for the sake of getting a better understanding of the evolution of the UDPGP gene family. Moreover, the expression profiles, motif and key amino acids, gene variation, 3D structures, and isoforms of UDPGPs in *O. sativa* were also surveyed for understanding the conserved function of the UDPGP genes.

## Results and Discussion

### Identification of UDP Glucose Pyrophosphorylase Genes in the Variety Species

To identify full-length UDPGP genes in different species, we searched the UDPGP genes using HMMER software (Potter et al., 2018). A total of 454 full-length primary protein sequences were identified in 76 organisms, including plants (58 species: 404 sequences), chlorophyte (seven species: 21 sequences), animals (six species: 16 sequences), fungi (three species: 11 sequences), kinetoplastid (one species: two sequences), and bacteria (one species: 0 sequence). The detailed information is in Supplementary Table 2.

### Phylogenetic Classification of the UDP Glucose Pyrophosphorylase Gene Family Into Three Major Clades

**FIGURE 1:**
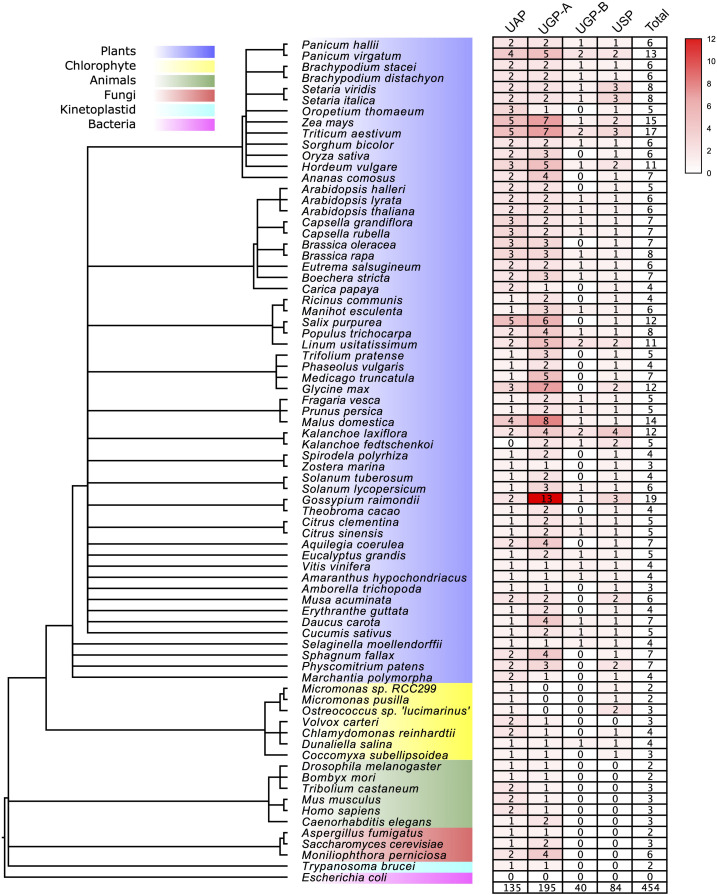
The UDP glucose pyrophosphorylase (UDPGP) genes were investigated in this study. The species tree was downloaded from the National Center for Biotechnology Information (NCBI) Taxonomy tree.

To study the origin and evolutionary history of the UDPGP genes in different species, we constructed a phylogenetic tree with the Maximum Likelihood (ML) method. The phylogenetic tree with gene names and bootstrap values (UFBoot/SH-aLRT/aBayes) is shown in Supplementary Figure 1. The topology of the ML tree showed that UDPGP genes were clustered into three clades, which were defined as UAP, UGP, and USP. Besides, The UGP clade could be divided into two subgroups, UGP-A and UGP-B. These results were similar to the OrthoFinder analysis results (Supplementary Table 3). The orthogroup analysis divided the gene family into seven orthogroups, and the first four largest orthogroups, OG1, OG2, OG3, and OG4, were corresponding with the subgroup UGP-A, UAP, USP, and UGP-B, respectively, while three minor orthogroups (OG5, OG6, and OG7) contained UAP and UGP-A members. Notably, no homologous UGP-B and USP members were identified in animals, fungi, and kinetoplastid. Furthermore, *Escherichia coli* does not contain any homologous genes belonging to UDPGP in this study, which is corresponding to that of previous studies (Kleczkowski et al., 2004; Führing et al., 2013). Then, we analyzed the distribution of each subgroup in the 58 plants (Figure 1 and Supplementary Table 2). The result showed that the numbers of UDPGP genes significantly varied among the major lineages of plants, ranging from the two members detected in chlorophytes including *Micromonas* sp. *RCC299* and *Micromonas pusilla* to the 19 sequences identified in *Gossypium raimondii*. In general, the numbers of UAP and USP genes were relatively stable among the plants, ranging from 0 to 5 (UAP) and 0 to 4 (USP), while the members in UGP (UGP-A and UGP-B) varied greatly, spanning from 0 to 14 (*G. raimondii*). Specifically, we did not find any full-length UAP genes in *Kalanchoe fedtschenkoi* or USP genes in *Volvox carteri*. In addition, *Micromonas* sp. *RCC299*, *Micromonas pusilla*, *and Ostreococcus* sp. *Lucimarinus* had no UGP (UGP-A or UGP-B) genes (Figure 1 and Supplementary Table 2).

### Physicochemical Features of UDP Glucose Pyrophosphorylase Gene Family

The calculated isoelectric point (pI) and molecular weight (MW) of each UDPGP sequence are shown in Supplementary Table 2. The average MWs of subgroups UAP, UGP-A, UGP-B, and USP were 56,234.91, 47,351.92, 93,678.22, and 66,044.22 Da, respectively. The results showed remarkable differences among the four subgroups, with subgroup UGP-A containing the smallest MW and subgroup UGP-B containing the heaviest MW. The pI for the UDPGP genes ranged from 4.33 to 9.99, implying a wide range of activity in microcellular environments. The subgroup UAP had more acidified pI with an average of 6.00 compared with the other subgroups, suggesting possible functional divergence of UAP (Supplementary Table 2). All these showed the conserved physicochemical features in UDPGP genes.

### Gene Structure Difference in the Three Clades

**FIGURE 2:**
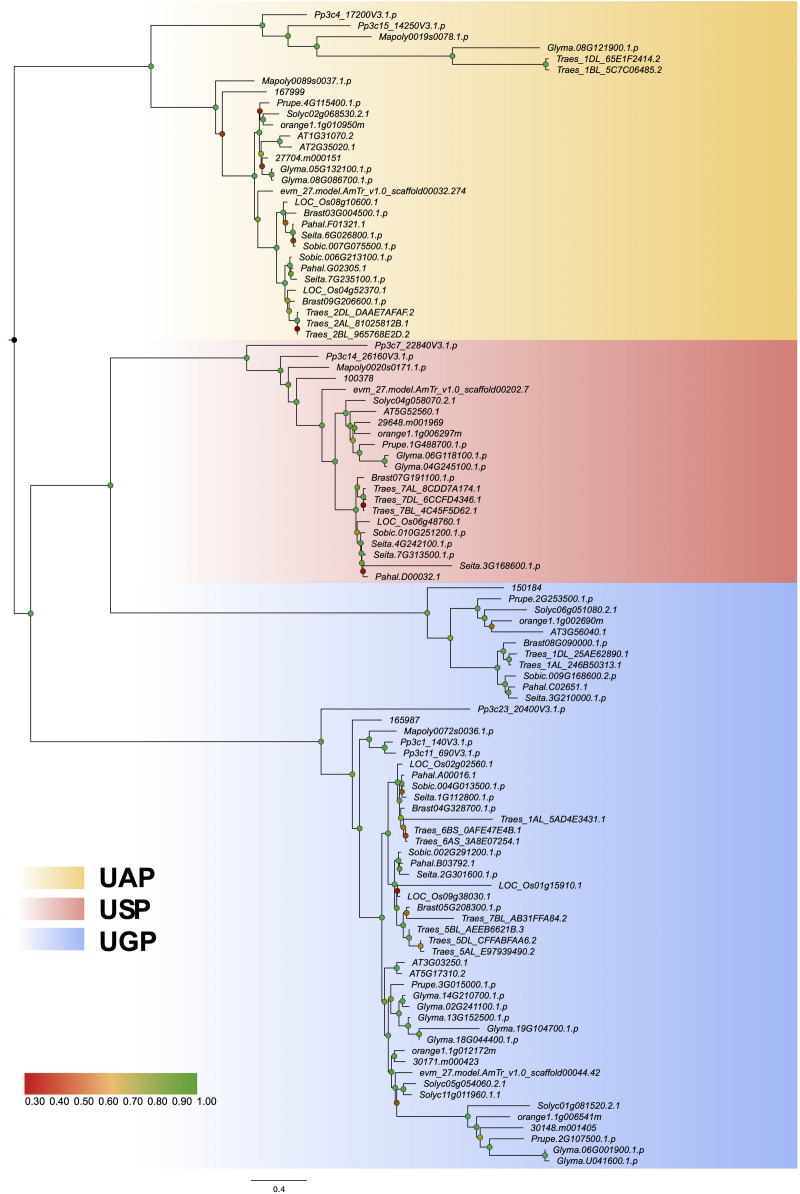
Maximum likelihood phylogenetic tree of UDP glucose pyrophosphorylases (UDPGPs). A maximum likelihood analysis phylogenetic tree illustrates the evolutionary relationships among UDPGP sequences from 16 species representing a wide variety of plant lineages and the ancestral homolog. The three phylogenetic clusters were designated as UDP-N-acetylglucosamine pyrophosphorylase (UAP; yellow), UDP-sugar pyrophosphorylase (USP; red), and UDP-glucose pyrophosphorylase (UGP; blue). Statistical support is shown in corresponding nodes at relevant clades according to the color of the label. Branch lengths in the tree are proportional to evolutionary distances between nodes, and the scale bar represents the number of inferred amino acid substitutions per site.

The loss or gain of introns leads to different gene structures, makes genes more complex, and acts as the foundation of gene evolution (Fedorova and Fedorov, 2003). Previous studies have shown that introns play an important biological role in regulating gene expression (Castillo-Davis et al., 2002; Le Hir et al., 2003). In the current study, we constructed a phylogenetic tree using protein sequences from 16 representative plant species (Figure 2) and analyzed the exon–intron structures (Supplementary Figure 2). The exon–intron organization of UDPGP genes was significantly different among the four subgroups, while the structure within each subgroup was conserved, indicating the conservative characteristics in the UDPGP gene family (Supplementary Figure 2). Besides, the numbers of exons in each subgroup are similar, ranging from 15 in UAP to 21 in UGP-A typically.

### The Divergence and Segmental Duplication of UDP Glucose Pyrophosphorylase Family

**FIGURE 3:**
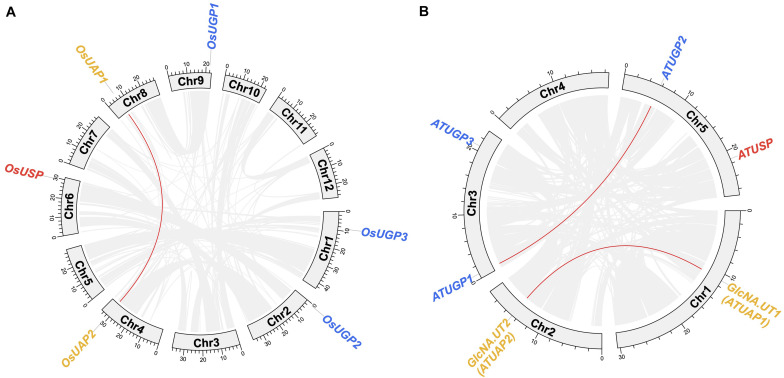
Chromosome distributions of UDP glucose pyrophosphorylases (UDPGPs). The chromosomal distributions of UDPGP genes in *Oryza sativa* **(A)** and *Arabidopsis thaliana* **(B)** are shown in the outer circle, where the numbers represent the chromosome length 10 Mb. The synteny and collinearity genes detected by MCScanX are connected by red arcs in the inner circle.

In the evolutionary history of the UDPGP gene family, the number of members was relatively stable, resulting in an elementary gene family. As shown in Figure 3A, six of 12 chromosomes contain UDPGP genes in *Oryza sativa*. Three *OsUGP* genes (*OsUGP1*, *OsUGP2*, *OsUGP3*) posited on chromosome 9, chromosome 2, chromosome 3, respectively. Synteny analysis of the UDPGP family in rice showed that collinearity blocks between UDPGP members only existed in clade UAP (*OsUAP1* and *OsUAP2*) and located on chromosome 8 and chromosome 4. Furthermore, the genome of *O. sativa* only contains a single *OsUSP* gene located on chromosome 6. The similarity of *OsUAP1* and *OsUAP2* indicated that these two genes originated from duplication. Among the five chromosomes in *Arabidopsis*, only chromosome 4 carried no UDPGP genes, and others had one or two UDPGP genes (Figure 3B). Chromosome 3 contained two *ATUGP* genes (*ATUGP1* and *ATUGP3*), and chromosome 5 contained *ATUSP* and *ATUGP2* genes at both arms. Besides, chromosome 1 and chromosome 2 separately hold *GlcNA.UT1* (*ATUAP1*) and *GlcNA.UT2* (*ATUAP2*) genes. In addition, we also detected duplication events, which were similar to *O. sativa*, in *A. thaliana*. The *ATUAP1* and *ATUAP2*, *ATUGP1* and *ATUGP2* were two paired collinearity genes detected by McScanX, while no tandem gene pairs were identified in both *O. sativa* and *A. thaliana*. All these results showed that the members of UDPGP genes were stable and the expansions of the UDPGP gene family were only caused by segmental duplication.

### Expression Patterns of UDP Glucose Pyrophosphorylase Genes in Different Tissues

**FIGURE 4:**
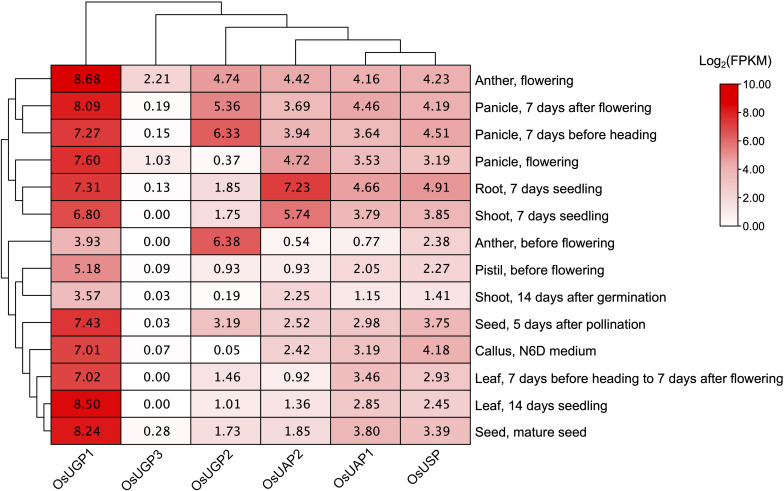
UDP glucose pyrophosphorylase (UDPGP) expression profiles in different tissues from *Oryza sativa*. The RNA sequencing expression data of UDPGPs from different tissues and developmental stages in *O. sativa* were downloaded from the Rice Expression Database and displayed as filled blocks from white to red.

The transcriptomic profile reflects the tissue-specific function. RNA sequencing data from Rice Expression Database (RED) were downloaded to analyze the UDPGP genes in *O. sativa* expression pattern in eight (anther, callus, leaf, panicle, pistil, root, seed, shoot) different tissues (Figure 4). The results showed that the expression level of *LOC_Os09g38030* (*OsUGP1*) was significantly higher than that of other gene members in the UDPGP family. Anther during flowering is the highest expressing tissue detected with *OsUGP1*. The *LOC_Os02g02560* (*OsUGP2*) and *LOC_Os01g15910* (*OsUGP3*) also belonged to the UGP clade in rice, whose expression levels were greatly lower than that in *OsUGP1*. For *OsUGP2*, anther (before and during flowering) and panicle (7 days before heading and 7 days after flowering tissues) in specific development stages exhibited higher expression levels than other tissues. Notably, *OsUGP3* showed almost no expression in the seven tissues. Besides, for the UAP clade *LOC_Os04g52370* (*OsUAP2*) and *LOC_Os08g10600* (*OsUAP1*), the expression patterns were similar to each other and the expression level of *OsUAP2* gene was slightly higher than *OsUAP1* in some specific tissues, such as root and shoot from 7-day seedlings. In addition, for the *OsUSP* gene, a single member of the rice USP clade, the expression profile was ubiquitous in all tissues with a relatively low level. These results suggest that rice UDPGP genes can be expressed in various tissues to perform their roles with different expression levels as needed.

### Expression Profiles of UDP Glucose Pyrophosphorylases Under Hormones, Abiotic, Non-metal, and Heavy Metal Stress

**FIGURE 5:**
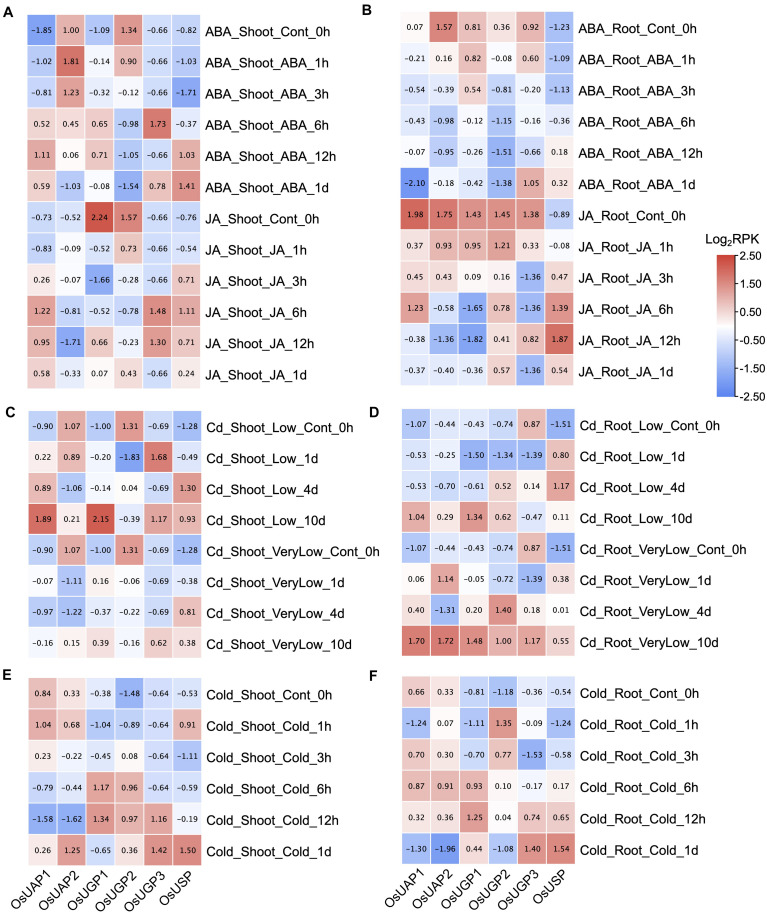
**(A–F)** UDP glucose pyrophosphorylase (UDPGP) expression profiles under abscisic acid (ABA), jasmonic acid (JA), cadmium, and cold treatment. Transcriptional expression changes of UDPGPs in *Oryza sativa* shoot under ABA (100 μM), JA (100 μM), low cadmium (1 μM CdSO_4_), very low cadmium (0.2 μM CdSO_4_), and cold (4**°**C) treatment were downloaded from the TENOR database and displayed as filled blocks from red (upregulated) to blue (downregulated). The scale on the right indicates the gene expression level transformed by log_2_(RPK). All the genes were normalized by columns.

Plant hormones, abscisic acid (ABA) and jasmonic acid (JA), are important regulatory factors involved in various biological processes, including cell death (Sreenivasulu et al., 2006; Reinbothe et al., 2009). The expression patterns of rice UDPGP genes under ABA and JA treatments are similar in both shoot (Figure 5A) and root (Figure 5B) tissues. Importantly, *OsUAP1*, *OsUGP3*, and *OsUSP* were generally upregulated in shoot tissue under the ABA and JA stress, and *OsUSP* was also upregulated in root tissue under the ABA and JA stress.

Cadmium (Cd) contamination has become a big issue in food safety, especially in rice (Liu et al., 2020). Under the very low Cd and low Cd treatments, the expression level of all six rice UDPGP genes were uniformly upregulated in root tissue except for *OsUGP3* (Figure 5D). The three genes (*OsUAP1*, *OsUGP1*, and *OsUSP*) also showed increased expression trend in shoot tissue (Figure 5C) under the same treatments. When stressed by cold, the four rice UDPGP genes (*OsUGP1*, *OsUGP2*, *OsUGP3*, and *OsUSP*) were raised both in shoot (Figure 5E) and root (Figure 5F).

Moreover, the expressions of UDPGP genes in *O. sativa* under abiotic (salinity, dry, flood, and osmotic) and non-metal [phosphate (P)] (Supplementary Figure 3) treatments were also investigated. In general, *OsUAP1* and *OsUSP* were found to be upregulated under dry, flood, and osmotic stresses of shoot, and *OsUSP* was also upregulated under flood and osmotic stresses of root. Moreover, *OsUAP2* and *OsUGP2* separately showed upregulated expression under dry stress of shoot and osmotic stress of root. No significant expression changes were identified for UDPGP genes under salinity and P treatment in both shoot and root tissues.

The expression data above together implied that rice UDPGP genes may be involved in environmental endurance.

### Conserved Motifs in UDP Glucose Pyrophosphorylase Family Members and the Key Amino Acids Affect Catalytic Activity

**FIGURE 6:**
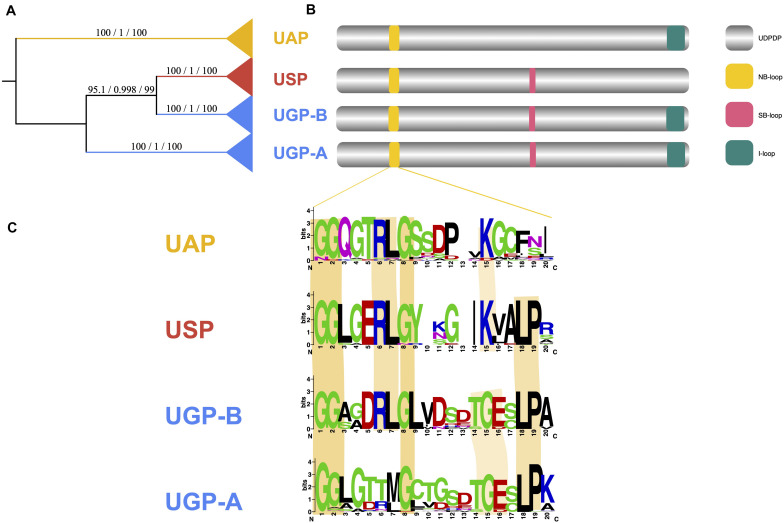
UDP glucose pyrophosphorylase (UDPGP) domain classification and conservation of functional motifs. **(A)** Phylogram showing the classification of the UDPGP gene family with the major clades labeled. **(B)** Typical UDPGP structure consists of an NB-loop, SB-loop, and I-loop with the approximate positions. **(C)** Conservation of the NB-loop functional motifs in different clades.

Phylogenetic analysis of the UDPGP homologs yielded three big different clades and four distinct subgroups (Figure 6A). In previous reports, UGP-A clade contained a nucleotide-binding loop (NB-loop) and substrate binding loop (SB-loop) at the active center and an insertion loop (I-loop) at the C-terminus site (Steiner et al., 2007; Führing et al., 2015; Chi et al., 2016; Figure 6B). For the UAP clade, only NB-loop and I-loop were reported (Peneff et al., 2001; Figure 6B). In the USP clade, NB-loop and SB-loop were described (Dickmanns et al., 2011; Figure 6B). Based on the NB-loop reported by Geisler et al. (2004), we identified the differences of the NB-loop in the three clades through multiple sequence alignment (MSA) (Figure 6C). The results showed that NB-loop was diverse in different subgroups. In general, all the NB-loops contained a start with “GGxG.”

Then, the motifs from 16 represented species were analyzed based on the primary protein sequences using MEME software. As shown in Supplementary Figure 4, motifs 1, 4, 13, and 14 existed in all subgroups, while motifs 20, 18, and 15 were unique sequences at the N-terminus in UAP, USP, and UGP-A subgroups, respectively. Besides, motif 14 was a common motif at the C-terminus in all UDPGP clades except the UAP clade, which carried extra motifs 5, 9, and 17. Furthermore, motifs 6, 16, and 19 were only located in the USP clade, while the UGP-A subgroup contained distinctive motifs 7, 8, 10, 12, and 14. In our study, we defined a motif 8 with “NPSIELGPEFKKVGNFLSRFKSIPSIVELDSLKVSGDVWFG” sequence, which overlapped with a previously reported motif “RFKSIPSI,” and this motif was proven to be an essential element for the phosphorylation and binding with 14-3-3 protein (Toroser et al., 1998; Cotrim et al., 2018). Moreover, motif 2 “KLAVLLLAG*G*LGT*R*LGCTGP*K*” displayed in all clades except UGP-B; this motif was corresponding to that of a previous study that reported a high essential signature motif “LX2GXGTX6PK” (Mio et al., 1998) and three amino acids (G, R, and K) were proven vital for the activity of the enzyme. All the above contributed to the form of the UDPGP gene family and diverged it into three large clades (UAP, USP, and UGP). Thus, the conservation of these additional motifs in their respective clades may play a key role in their functional specificity.

In *OsUAP1* mutant *osuap1*, guanine (G) was replaced by thymine (T) at the position of 712 bp in the CDS, resulting in the 238th amino acid changed from glycine (Gly) to cysteine (Cys) and thus lost function of UAP enzymatic activities for the *OsUAP*1 protein (Wang et al., 2015; Supplementary Figure 5). This key amino acid site is located in motif 2, which exhibited in all clades except UGP-B, and this site might be involved in the uridine recognition region (Wang-Gillam et al., 2000). Some single amino acid and fragment deletion mutations were performed to study the key amino acid of UDPGPs. In the UAP clade, 14 site-directed mutants were used to study the key amino acid affecting the catalytic activity, including *Giardia intestinalis* (Mok and Edwards, 2005), *Homo sapiens* (Wang-Gillam et al., 2000; Peneff et al., 2001), *Saccharomyces cerevisiae* (Mio et al., 1998), and *Aspergillus fumigatus* (Raimi et al., 2020; Supplementary Figure 6 and Supplementary Table 4). In *G. intestinalis* (Mok and Edwards, 2005), when G108 (corresponding to G125 in *OsUAP1*) and G210 (corresponding to G236 in *OsUAP1*) were substituted by alanine, the enzymatic activity of UAP was significantly reduced. In *H. sapiens* (Wang-Gillam et al., 2000; Peneff et al., 2001), R115, P220, G222, G224, Y227, and G111 were replaced by alanine to study the catalytic property. The results showed that R115, G222, G224, and G111 were key amino acids to maintain the activity of the enzyme, corresponding to R129, G236, G238, and G125 in *OsUAP1*. Besides, the G111 and R115 were located in the NB-loop as well as motif 2, indicating the importance of the sequence. In *Saccharomyces cerevisiae* (Mio et al., 1998), three amino acids were studied, including G112, R116, and K123, which equated to G125, R129, and K136 in *OsUAP1*. The enzymatic activity was severely diminished when these three sites were replaced by alanine. In *A. fumigatus*, five site mutations were performed to study the key amino acids in *AfUAP* gene (Raimi et al., 2020). Three (K148, Y330, and K437) of them showed a key role in impacting Km value of UTP or GlcNAc-1P, corresponding respectively to K136, Y320, and K416 in *OsUAP1* in rice. Several mutants in *Hordeum vulgare* (Martz et al., 2002; Meng et al., 2009a), *Solanum tuberosum* (Katsube et al., 1991), *Cricetulus griseus* (Flores-Díaz et al., 1997), and *H. sapiens* (Chang et al., 1996), including single amino acid and fragment deletion, were used to study the key amino acids of UGP (Supplementary Figure 7). In *H. vulgare* (Martz et al., 2002; Meng et al., 2009a), G91, C99, L117, I118, V119, K127, K128, L135, L136, L137, Y192, and K260 were key amino acids to provide the activity of proteins, which corresponded to G88, C96, L114, I115, V116, K124, K125, L132, L133, L134, Y189, and K257 in *OsUGP1*. And when K183, K332, K405 were replaced by alanine, the activity was not greatly impacted in *HvUGP* (HORVU5Hr1G087810.2). These three sites corresponded to the K180, K329, and K402 in *OsUGP1*. In addition, the authors trimmed different lengths of N and C terminals to study the key role in regulating the activity. Results showed that the majority (Ncut-21, Ncut-27, Ccut-8, Ccut-67, and Ccut-101) largely reduced the activity except for the cut 32 amino acids in C terminal, indicating the importance of N and C terminals for catalytic activity. In *S. tuberosum* (Katsube et al., 1991), five site mutants were studied through substituting with glutamine, including K263, K329, K367, K409, and K410. Among them, K263 and K367 were two important sites to keep the activity of UGP in *S. tuberosum*, which corresponded to K257 and K361 in *OsUGP1*. When G115 was replaced by aspartic acid (D) in *Cricetulus griseus* (Flores-Díaz et al., 1997), the activity of UGP was largely affected, which corresponded to G88 in *OsUGP1*. In *H. sapiens* (Chang et al., 1996), eight site mutants were developed including C123, W218, H266, W333, R389, R391, R422, and R445. Among them, C123, W333, R389, R391, and R422, corresponding to C96, W299, R354, R356, and R387 in *OsUGP*, were key amino acids for sustaining the activity. Among these, G115 in *CgUAP* was mapped to NB-loop, which corresponded to motif 2 assigned by MEME. And K236 in *StUGP* was a key amino acid located in SB-loop, which was in agreement with motif 3. The rest of the amino acids were located in motifs 4, 7, 8, and 11. Furthermore, no visible differences of activity were described when K183, K332, and K405 in *HvUGP* (Martz et al., 2002; Meng et al., 2009a) and W218, H266, and R445 in *HsUGP* (Chang et al., 1996) were substituted by other amino acids, indicating that these sites were not key amino acids in UGP protein. In addition, these four amino acids were not located in any motif assigned by MEME. The only one study reported on the key amino acid of USP was from *Leishmania major* (Prakash et al., 2019). V330, F383, and V199 were key sites for the activity of USP in *LsUSP*, corresponding to Q353, F405, and V239 in *OsUSP* (Supplementary Table 4 and Supplementary Figure 8). These sites were located in motifs 13, 16, and 19. Moreover, we compared the key amino acids, important loops (NB-loop, SB-loop, and I-loop), and motifs from MEME. The results showed that the amino acids in the loops or the motifs more likely contributed to the catalytic activity of UDPGPs.

### Most UDP Glucose Pyrophosphorylase Genes Are Conserved in Natural Rice Variant

Previous studies showed that the mutants of UDPGP genes lead to aborting of enzymatic activity, resulting in cell death (Tresse et al., 2008). Besides, the family numbers of UAP and USP clades are conserved, and many species contained only two UAP genes or a single USP gene. To address the question if the amino acid sequences are conserved in UDPGP genes, we scanned the protein sequences of UDPGPs in 4,726 rice accessions in RiceVarMap^1^. A total of 4, 2, 5, 2, 23, and 7 gene variants for *OsUAP1*, *OsUAP2*, *OsUGP1*, *OsUGP2*, *OsUGP3*, and *OsUSP* were identified, respectively (Table 1). Furthermore, 32 of 43 variation sites located in the non-motif region, and only 11 posited on the motif sites. Among the 11 variants, only two of them showed a relatively higher proportion in nature. The 502nd amino acid (in Motif 8) changed from Ser to Asn in *OsUGP3* with 40.40% and the 462nd amino acid changed (in Motif 6) from Ser to Ala in *OsUSP* with 28.30% (Table 1). In the previous study, one amino acid in Motif 8 was mutant in wheat, and the result showed it only slightly lowered the enzymatic activity (Meng et al., 2009a). In addition, the number of *OsUGP3* variants was greater than other members (Table 1), but the expression levels of *OsUGP3* were much lower than other members (Figure 4), which may indicate that this gene is under evolution. All above suggest that most UDPGP genes are conserved in the evolution.

**TABLE 1:** Natural variant of UDPGP genes in rice.

Gene name	LOC name	SNP position	Primary allele	Secondary allele	Primary allele frequency	Amino acid mutation and position	snpEff annotation	Motif
*OsUAP1*	*LOC_Os08g10600.1*	210	G	T	98.70%	Cys70Phe	missense_variant	motif_20
*OsUAP1*	*LOC_Os08g10600.1*	570	A	T	99.90%	Lys190Asn	missense_variant	no
*OsUAP1*	*LOC_Os08g10600.1*	726	G	A	99.80%	Ala242Thr	missense_variant	motif_1
*OsUAP1*	*LOC_Os08g10600.1*	855	G	T	99.90%	Lys285Asn	missense_variant	motif_14
*OsUAP2*	*LOC_Os04g52370.1*	846	G	T	99.40%	Phe282Leu	missense_variant	no
*OsUAP2*	*LOC_Os04g52370.1*	39	ACCGCCG	ACCGCCG CCG	48.90%	Ala13dup	inframe_insertion	no
*OsUGP1*	*LOC_Os09g38030.1*	12	G	A	63.90%	Thr4Ala	missense_variant	no
*OsUGP1*	*LOC_Os09g38030.1*	117	G	A	99.70%	Ser39Asn	missense_variant& splice_region_variant	motif_15
*OsUGP1*	*LOC_Os09g38030.1*	153	G	T	99.40%	Gln51His	missense_variant	motif_15
*OsUGP1*	*LOC_Os09g38030.1*	1,311	C	G	99.20%	Leu437Val	missense_variant	motif_14
*OsUGP1*	*LOC_Os09g38030.1*	1,386	C	G	60.60%	Asp462His	missense_variant& splice_region_variant	no
*OsUGP2*	*LOC_Os02g02560.1*	1218	C	G	94.30%	Gly406Arg	missense_variant	motif_8
*OsUGP2*	*LOC_Os02g02560.1*	186	T	G	60.60%	Ala62Glu	missense_variant	no
*OsUGP3*	*LOC_Os01g15910.1*	36	C	T	89.80%	Pro12Leu	missense_variant	no
*OsUGP3*	*LOC_Os01g15910.1*	51	G	A	97.80%	Ala17Thr	missense_variant	no
*OsUGP3*	*LOC_Os01g15910.1*	102	G	A	58.10%	Gly34Glu	missense_variant	no
*OsUGP3*	*LOC_Os01g15910.1*	108	C	T	58.00%	Ala36Val	missense_variant	no
*OsUGP3*	*LOC_Os01g15910.1*	186	A	G	43.50%	Arg62Gly	missense_variant	no
*OsUGP3*	*LOC_Os01g15910.1*	192	C	T	78.30%	Pro64Leu	missense_variant	no
*OsUGP3*	*LOC_Os01g15910.1*	207	A	G	44.30%	Lys69Glu	missense_variant	no
*OsUGP3*	*LOC_Os01g15910.1*	234	A	T	50.60%	Val78Asp	missense_variant	no
*OsUGP3*	*LOC_Os01g15910.1*	267	A	C	59.70%	Asp89Ala	missense_variant	no
*OsUGP3*	*LOC_Os01g15910.1*	450	G	A	89.90%	Gly150Ser	missense_variant	no
*OsUGP3*	*LOC_Os01g15910.1*	510	A	G	50.30%	Val170Met	missense_variant	no
*OsUGP3*	*LOC_Os01g15910.1*	858	A	G	89.80%	Asn286Ser	missense_variant &splice_region_variant	no
*OsUGP3*	*LOC_Os01g15910.1*	1,425	G	A	59.60%	Gly475Asp	missense_variant	no
*OsUGP3*	*LOC_Os01g15910.1*	1,503	C	A	59.60%	Asp501Glu	missense_variant	no
*OsUGP3*	*LOC_Os01g15910.1*	1,506	G	A	59.60%	Ser502Asn	missense_variant	motif_8
*OsUGP3*	*LOC_Os01g15910.1*	1,572	G	A	99.90%	Asp524Asn	missense_variant	motif_8
*OsUGP3*	*LOC_Os01g15910.1*	1,803	T	A	97.30%	Asp601Glu	missense_variant	no
*OsUGP3*	*LOC_Os01g15910.1*	1,836	A	G	50.40%	Arg612Gln	missense_variant	no
*OsUGP3*	*LOC_Os01g15910.1*	1,893	A	G	99.90%	Asp631Gly	missense_variant	no
*OsUGP3*	*LOC_Os01g15910.1*	1,965	G	T	50.40%	Leu655Arg	missense_variant	no
*OsUGP3*	*LOC_Os01g15910.1*	2,088	A	G	59.50%	Ser696Gly	missense_variant	no
*OsUGP3*	*LOC_Os01g15910.1*	2,133	A	C	50.40%	Lys711Asn	missense_variant	no
*OsUGP3*	*LOC_Os01g15910.1*	2,166	C	T	97.30%	Ala722Val	missense_variant	no
*OsUSP*	*LOC_Os06g48760.1*	1,569	A	G	91.90%	Leu523Pro	missense_variant	no
*OsUSP*	*LOC_Os06g48760.1*	1,386	A	C	71.70%	Ser462Ala	missense_variant	motif_6
*OsUSP*	*LOC_Os06g48760.1*	1,353	C	T	70.50%	Thr451Ala	missense_variant	no
*OsUSP*	*LOC_Os06g48760.1*	468	T	A	97.30%	Tyr156Phe	missense_variant	motif_20
*OsUSP*	*LOC_Os06g48760.1*	123	T	C	97.10%	Lys41Glu	missense_variant	no
*OsUSP*	*LOC_Os06g48760.1*	90	C	T	97.30%	Arg30Gln	missense_variant	no
*OsUSP*	*LOC_Os06g48760.1*	18	C	T	84.90%	Asp6Asn	missense_variant	no

*SNP, single-nucleotide polymorphism; UDPGP, UDP glucose pyrophosphorylase.*

### Alternative Splicing Events Affect Protein Activities Among the UDP Glucose Pyrophosphorylase Gene Family

**FIGURE 7:**
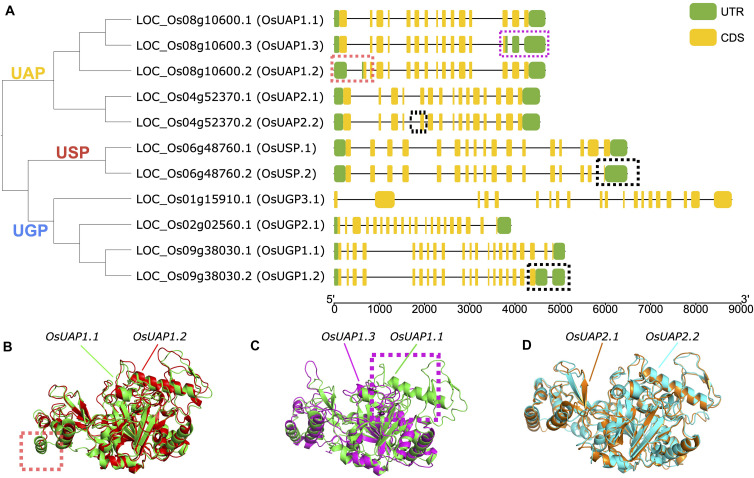
Alternative splicing of UDP glucose pyrophosphorylases (UDPGPs) in *Oryza sativa*. **(A)** Gene structure and phylogenetic tree of UDPGP genes in *O. sativa*, different alternative splicing events were marked with red (OsUAP1.2), purple (OsUAP1.3), or black dashed rectangles. **(B)** Comparison of the 3D protein structure of OsUAP1.1 (green) and OsUAP1.2 (red). **(C)** Comparison of the 3D protein structure of OsUAP1.1 (green) and OsUAP1.3 (purple). **(D)** Comparison of the 3D protein structure of OsUAP2.1 (orange) and OsUAP2.2 (blue).

Alternative splicing events are important posttranscriptional regulatory mechanisms, which could reduce the enzyme activities or completely abolish the activity (Kelemen et al., 2013). Here we studied the alternative splicing events of the UDPGP gene family in *O. sativa*. Four (*OsUAP1*, *OsUAP2*, *OsUSP*, and *OsUGP1*) of six UDPGP genes carried more than one transcript sequence from the rice annotation file. The *OsUAP1* gene had three different types of mRNA sequences, *OsUAP1.1*, *OsUAP1.2*, *and OsUAP1.3*. The *OsUAP1.1* transcript was the longest form with 489 amino acids, while the *OsUAP1.2* transcript lacked exon sequence near 5’ untranslated region (5’ UTR), and the *OsUAP1.3* transcript lost exons at 3’ UTR (Figure 7A). The *OsUAP2* gene had two similar transcripts with a difference of six nucleotides at the end of the fifth exon (Figure 7A and Supplementary Figure 9). The *OsUSP* gene and the *OsUGP1* gene both had two transcripts with the exon differences closing to the 3’ UTR.

To compare the structure differences for these gene isoforms, we predicted the 3D structures of proteins using I-TASSER. The results showed that the spatial structures among the three *OsUAP1* isoforms were similar, while *OsUAP1.2* (Figure 7B) and *OsUAP1.3* (Figure 7C) lacked some folding due to the deficiency of exons at 5’ UTR and 3’ UTR. And for *OsUAP2* isoforms, the predicted tertiary structures of *OsUAP2.1* and *OsUAP2.2* were almost the same (Figure 7D) due to the tiny difference between *OsUAP2.1* and *OsUAP2.2* variants, with *OsUAP2.2* discarding two amino acids compared to *OsUAP2.1* (Supplementary Figure 9). In addition, the structures of *OsUGP1.1*, *OsUGP1.2*, *OsUSP.1*, and *OsUSP.2* were also predicted to compare differences in the spatial structures (Supplementary Figure 10). The results showed that the N and center domains were similar, while the C terminal was diverse because of the lack of sequences in *OsUGP1.2* and *OsUSP.2*.

**FIGURE 8:**
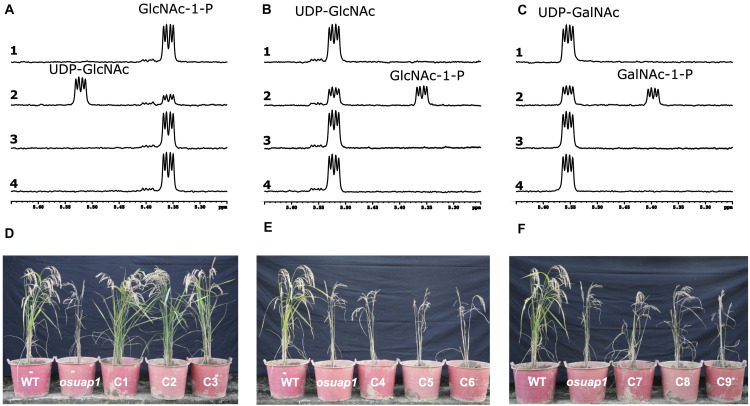
*In vitro* and *in vivo* activity of isoforms from OsUAP1. Enzymatic activities of three isoforms of OsUAP1 based on ^1^H-nuclear magnetic resonance (^1^H-NMR). **(A)** Forward activity: UTP + GlcNAc-1-P → UDP-GlcNAc + PPi. **(B)** Reverse activity: UDP-GlcNAc + PPi → GlcNAc-1-P + UTP. **(C)** Reverse activity: UDP-GalNAc + PPi → GalNAc-1-P + UTP. **(A–C)** Line 1, glutathione S-transferase (GST) control. Line 2, the protein of OsUAP1.1. Line 3, the protein of OsUAP1.2. Line 4, the protein of OsUAP1.3. **(D)** The phenotype of wild-type (WT), mutant (osuap1), and three independent complementary transgenic lines overexpressing OsUAP1.1. **(E)** The phenotype of WT, mutant (osuap1), and three independent complementary transgenic lines overexpressing OsUAP1.2. **(F)** The phenotype of WT, mutant (osuap1), and three independent complementary transgenic lines overexpressing OsUAP1.3.

To understand if the alternative splicing events in UDPGP genes affect the catalytical characteristics, we performed the enzymatic activity analysis in the UAP clade. ^1^H-nuclear magnetic resonance (^1^H-NMR) spectroscopy was used to record the enzymatic reaction of *OsUAP1.1*, *OsUAP1.2*, and *OsUAP1.3 in situ*. In the time-gradient enzymatic progression at 60 min, forward conversion of GlcNAc-1-P (5.36 ppm) to UDP-GlcNAc (5.52 ppm) was observed with *OsUAP1.1* (Figure 8A, line 2), but not with the glutathione S-transferase (GST) control (Figure 8A, line 1), *OsUAP1.2* (Figure 8A, line 3), and *OsUAP1.3* (Figure 8A, line 4). Besides, the reverse conversion of UDP-GlcNAc (5.52 ppm) to GlcNAc-1-P (5.36 ppm) was identified with *OsUAP1.1* (Figure 8B, line 2), but not with the GST control (Figure 8B, line 1), *OsUAP1.2* (Figure 8B, line 3), and *OsUAP1.3* (Figure 8B, line 4). Furthermore, *OsUAP1.1* could also catalyze the reverse conversion of UDP-GalNAc (5.55 ppm) to GalNAc-1-P (5.39 ppm) (Figure 8C, line 2), whereas GST (Figure 8C, line 1), *OsUAP1.2* (Figure 8C, line 3), and *OsUAP1.3* (Figure 8C, line 4) could not perform this reaction. Furthermore, we also conducted the same reaction in the *OsUAP2* gene. The *OsUAP2* gene was another member of the UAP clade in rice, which contained two isoforms (*OsUAP2.1* and *OsUAP2.2*). The results showed that both *OsUAP2.1* and *OsUAP2.2* could catalyze the reaction from GlcNAc-1-P to UDP-GlcNAc, UDP-GlcNAc to GlcNAc-1-P, and UDP-GalNAc to GalNAc-1-P (Supplementary Figures 11A–C).

Losing UAP enzymatic activity for *OsUAP1* induces early leaf senescence (Wang et al., 2015). Actually, all three alternatives (*OsUAP1.1*, *OsUAP1.2*, and *OsUAP1.3*) were mutated, corresponding to the 238, 203, and 238 in amino acid sequences (Gly to Cys), respectively (Supplementary Figure 5). Accordingly, this mutant *osuap1* was used to identify the function of *OsUAP1.1*, *OsUAP1.2*, *OsUAP1.3*, *OsUAP2.1*, and *OsUAP2.2*. Results showed that the *osuap1* exhibited early leaf senescence, and overexpressing *OsUAP1.1* in *osuap1* could restore the healthy leaf (C1, C2, and C3 in Figure 8D) but not with *OsUAP1.2* (C4, C5, C6 in Figure 8E) and *OsUAP1.3* (C7, C8, C9 in Figure 8F). All the above indicated the key role of the N and C terminals in maintaining the activity of UAP. The UAP protein would lose its function without N or C terminal, which corresponded to a previous study on UGP protein (Supplementary Figure 7; Meng et al., 2009a). Then, we test whether the *OsUAP2.1* and *OsUAP2.2* could restore the *osuap1* mutant. The results showed that both the *OsUAP2.1* (Supplementary Figure 11D) and *OsUAP2.2* (Supplementary Figure 11E) also complemented the function of *OsUAP1.1*, making the leaves of *osuap1* grow normally without early leaf senescence.

The above results illustrated that the loss of N and C terminals in UDPGP gene isoforms did not affect the overall 3D structures, but these N- and C-terminal sequences may be important for the UDPGP gene isoforms to maintain or change their enzymatic activity. The molecular mechanisms for the existence of inactive UDPGP gene isoforms through alternative splicing are not clear. We proposed the following speculations: (1) the inactive UDPGP gene isoforms are mistake alternative splicing; (2) the inactive UDPGP gene isoforms are under evolution for new functions; (3) the inactive UDPGP gene isoforms have effective but unknown functions, for example, competing with active isoforms to bind the enzymatic substrates to regulate the enzymatic activities of active isoforms; (4) lack of the C terminal could reduce the spatial block of the UDPGP, resulting in forming an inactive dimer in the plant (Decker et al., 2012). All above represent potential regulating mechanisms of alternative splicing isoforms of UDPGP in the plant, and further experiments are needed to validate the hypothesis.

## Conclusion

In the present study, the phylogenetic tree of the UDPGP gene family from 76 organism lineages divided this gene family into three clades, including UAP, UGP, and USP, and the UGP could be additionally separated into two subclades, UGP-A and UGP-B. This result was also supported by the diverse physicochemical features, gene structures, and motifs in different clades. Through scanning the UDPGP gene members in 76 species, we found that the number of the UGP-A is more variable among the clades, while UGP-B, UAP, and USP showed relatively conserved members, indicating the important role of these UDPGP genes. UDPGP genes significantly respond to cadmium, cold, ABA, and JA stresses in the shoot or root tissues in rice, while they do not exhibit obvious feedback to salinity, dry, flood, osmotic, and phosphate stimulation. Through MSA, we identified the key amino acids regulating the enzymatic activities of the UDPGP proteins, and many of them located in the NB-loop, SB-loop, and conserved motifs, demonstrating the key role of these structures. Alternative splicing may be a key mechanism to regulate the enzymatic activity of UDPGP genes. In the current study, *in vitro* enzymatic experiments showed that the OsUAP1 lost its catalytic activity without the N (OsUAP1.2) or C (*OsUAP1.3*) terminal, while *OsUAP2.1* and *OsUAP2.2* both maintained their catalytic activity with complete N and C terminals. The same results were also proven in *in vivo* transgenic experiments, where the early senescence phenotype of the mutant *osuap1* could be rescued through overexpressing *OsUAP1.1*, *OsUAP2.1*, and *OsUAP2.2*, but not with *OsUAP1.2* and *OsUAP1.3*. All above provide new insights into the evolution and function of the UDPGP gene family, which may lay a foundation to further investigate their molecular regulatory mechanisms in the plant.

## Materials and Methods

### Data Sources and Sequence Retrieval

Protein sequences, transcript sequences, genomic sequences, and GFF annotation files of 58 green plants, seven chlorophytes, six animals, three fungi, one kinetoplastid, and one bacterium were downloaded from Phytozome^2^ and Ensembl website^3^ (Supplementary Table 1). UDPGP homologs were identified by the following steps: (1) A hidden Markov model (HMM) of UDPGP (ACC: PF01704.19) was downloaded from the Pfam website. (2) The UDPGP HMM was used to direct HMMSEARCH with the parameters *E*-value < 0.1. (3) Pfam-A.hmm was a manually corrected database and was downloaded from the Pfam website (April 18, 2020). Hmmscan software was used to identify the UDPGP domain with default parameters, and a total of 454 non-redundant primary protein sequences were retrieved for further analysis.

### Multiple Sequence Alignment and Phylogenetic Analysis

To explore the phylogenetic relationships of the UDPGP genes in the plant, animal, and microbial lineages, 454 full-length non-redundant primary protein sequences were used to perform MSA analysis using MAFFT V7.271 (Katoh and Standley, 2013) with default parameters. The MSA was submitted to IQ-TREE v2.1.1 (Minh et al., 2020) to be tested for the best substitution model, and the model with the lowest Bayesian Information Criterion (BIC) was selected as the best model (LG + I + G). Then, the phylogenetic tree was inferred by the ML method. We measured branch supports using the Ultrafast Bootstrap (UFBoot) algorithm with 1,000 replicates, the SH-aLRT, and approximate transformation Bayes test (aBayes). The tree was visualized using Interactive Tree of Life (iTOL)^4^ (Letunic and Bork, 2019) and FigTree v1.4.4^5^.

### Gene Structure, Sequence Motif Analysis, and Physicochemical Features

The coding sequence (CDS) information of the 105 full-length primary protein sequences from 16 representative plants was retrieved from the GFF annotation files and submitted to the TBtools (Chen et al., 2018) to visualize the exon–intron organization of UDPGP genes in representative species. Motif analysis was performed using MEME suite v5.3.0 (Bailey et al., 2009), which scans for motifs recurring in a set of sequences. Motif analysis was carried out using the MEME server^6^, keeping the width of the motif at 6–50 amino acids, the number of motifs was 20, and the other parameters set to default. The gene structure and conserved motif patterns were visualized by the TBtools (Chen et al., 2018). The pI/MW tool from ExPASy (Gasteiger et al., 2005) was used to compute the theoretical pI and MW of each sequence.

### Transcriptomic Analyses

The UDPGP gene expression data of *O. sativa* were downloaded from the Rice Expression Database^7^ and TENOR database^8^. The expression abundance with different treatments was transformed by log_2_RPK and visualized using TBtools.

### Orthogroup Generation

All full-length sequences from the 16 species (*Amborella trichopoda*, *A. thaliana*, *Brachypodium stace*, *Citrus sinensis*, *Glycine max*, *Marchantia polymorpha*, *O. sativa*, *Panicum hallii*, *Physcomitrella patens*, *Prunus persica*, *Ricinus communis*, *Selaginella moellendorffii*, *Setaria italica*, *Solanum lycopersicum*, *Sorghum bicolor*, and *Triticum aestivum*), including basal angiosperm, spikemoss, mosses, liverwort, monocots, and eudicots were selected to study the evolution of the UDPGP gene family (Supplementary Table 1). Orthologous genes were generated by OrthoFinder v2.2.7 (Emms and Kelly, 2015) with default parameters. In total, seven orthogroups were identified across the 16 species, and 105 (100%) of the input genes were assigned to orthogroups.

### Collinearity and Analysis

The collinearity of *O. sativa* and *A. thaliana* was detected with MCScanX (Wang et al., 2012). The result was visualized using TBtools (Chen et al., 2020).

### Evolutionary Expansion of UDP Glucose Pyrophosphorylase Gene Family

To understand and infer the evolutionary expansion history of the UDPGP family, we used a total of 105 full-length homologs from the 16 species.

### 3D Structure Modeling

The I-TASSER (Yang et al., 2014) was used to model the structures of all *O. sativa* UDPGP genes, including *OsUAP1*, *OsUAP2*, *OsUGP1*, *OsUGP2*, *OsUGP3*, and *OsUSP*. I-TASSER generates simulated protein structures depending on the pairwise structure similarity. Then, we selected the top models based on the C-score as representative structures. The 3D structures of proteins were visualized in PyMOL software.

### Enzymatic Reaction Experiments for OsUAP Isoforms Examined by ^1^H-Nuclear Magnetic Resonance Analysis

The GST gene fusion constructs of UAP isoforms were generated. The full-length CDS of the *OsUAP1.1*, *OsUAP1.2*, and *OsUAP1.3* isoforms were specifically amplified using primers *GST-OsUAP1.1* (F: *cgGGATCC*atggcggagatcgtggtggc, R: *cgGAATTC*ctaaaatgaaatctcactcggtgc), *GST-OsUAP1.2* (F: *cgGG ATCC*atggatgtacacagcc, R: *cgGAATTC*ctaaaatgaaatctcactcggtgc), and *GST-OsUAP1.3* (F: *cgGGATCC*atggcggagatcgtggtggc, R: *cgGAATTC*tcaagctttaagcctgccgtg). The full-length CDS of the *OsUAP2.1* and *OsUAP2.2* isoforms were amplified using primers *GST-OsUAP2* (F: *cgGGATCC*atgaaggagatagtggttgggtcg, R: *cgGAATTC*ctagaaggaaatctcactcggcg). PCR products were inserted into pGEX-6P-1 using the restriction enzyme sites *Bam*HI and *Eco*RI. Then, the recombinant vectors were transferred into *E. coli* DH5α and sequenced to check if the constructions were correct. Expression and purification of the fused GST-UAP isoforms were performed using the same method as described (Wang et al., 2015). The reaction of enzymatic activities of UAPs were performed as described previously (Wang et al., 2015), with 0.5 μg purified GST-UAP recombinant proteins used in the reaction mixture. Examination of GlcNAc-1-P/GalNAc-1-P and UDP-GlcNAc/UDP-GalNAc was performed by ^1^H-NMR as described (Zhang et al., 2011; Wang et al., 2015). Data acquisition started at 60 min respectively after the addition of an enzyme to the reaction mixture.

### Transgenic Experiments

The overexpression vectors of *UAP* gene isoforms were constructed. The full-length CDSs of the *OsUAP1.1*, *OsUAP1.2*, and *OsUAP1.3* isoforms were specifically amplified using primers *OsUAP1.1*-OE (F: *cagtGGTCTCa gttg*atggcggagatcgtggtggc, R: *cagtGGTCTCaagag*ctaaaatgaaatctca ctcg), *OsUAP1.2*-OE (F: *cagtGGTCTCagttg*atggatgtacacagcccact, R: *cagtGGTCTCaagag*ctaaaatgaaatctcactcg), and *OsUAP1.3*-OE (F: *cagtGGTCTCagttg*atggcggagatcgtggtggc, R: *cagtGGTCTC aagag*ctaaaatgaaatctcactcg). The full-length CDSs of the *OsUAP2.1* and *OsUAP2.2* isoforms were amplified using primer *OsUAP2*-OE (F: *cagtGGTCTCagttg*atgaaggagatagtggttgg, R: *cagtGGTCTCaagag*ctagaaggaaatctcactcg). PCR products were inserted into the binary vector pBWA(V)BU (reconstructed from pCAMBIA3300) using *Bsa*I sites for the digesting-link one-step reaction. The recombinant vectors were transferred into *E. coli* DH5α and sequenced to check if the constructions were correct. Correct vectors were introduced into *Agrobacterium tumefaciens* EHA105 and then transformed into the *osuap1* calli. Positive transgenic plants were confirmed using the *phosphinothricin* solution (20 mg/L) screening and then cultivated under natural conditions.

## Data Availability Statement

The original contributions presented in the study are included in the article/Supplementary Material, further inquiries can be directed to the corresponding author/s.

## Author Contributions

ZW designed the research. SL, HuZ, QW, CL, and ZW performed the research and analyzed the data. SL, HuZ, and ZW wrote the manuscript. TL, ZP, YL, HoZ, JL, YH, and ZW revised the manuscript. All authors contributed to the article and approved the submitted version.

## Conflict of Interest

The authors declare that the research was conducted in the absence of any commercial or financial relationships that could be construed as a potential conflict of interest.

## Footnotes

1 http://ricevarmap.ncpgr.cn/

2 https://phytozome-next.jgi.doe.gov/

3 http://uswest.ensembl.org/index.html

4 https://itol.embl.de/login.cgi

5 http://tree.bio.ed.ac.uk/software/figtree/

6 http://meme-suite.org/tools/meme

7 http://expression.ic4r.org/

8 https://tenor.dna.affrc.go.jp/

## Supplementary Material

Supplementary Figure 1 Maximum likelihood tree of 75 species generated by IQ-Tree and branch supports using ultrafast bootstrap approximation/SH-aLRT/aBayes methods. UAP, UGP, and USP were marked in yellow, blue, and red, respectively.

Supplementary Figure 2 Gene structures of 105 UDPGPs from 16 species. Phase 0 intron doesn’t interrupt a codon, phase 1 intron interrupts a codon between the 1st and 2nd bases, and phase 2 intron interrupts a codon between the 2nd and 3rd bases.

Supplementary Figure 3 Expression profile of UDPGPs of the shoot and root tissues under salinity (150 mM NaCl), dry (grown without medium), flood (completely submerged in medium), osmotic (0.6 M Mannitol), and P (3 mM KH_2_PO_4_) treatment from *Oryza sativa*. The scale on the right indicates the gene expression level transformed by log_2_(RPK). All the genes were normalized by columns.

Supplementary Figure 4 Motif architectures of 105 UDPGPs from 16 species.

Supplementary Figure 5 CDS alignment of three isoforms (OsUAP1.1, OsUAP1.2, and OsUAP1.3) from OsUAP1.

Supplementary Figure 6 Key amino acids affect UAP catalytic activities. The key amino acids were marked by black triangles. The black squares mean the deletion sites.

Supplementary Figure 7 Key amino acids affect UGP catalytic activities. The key amino acids were marked by dark triangles.

Supplementary Figure 8 Key amino acids affect USP catalytic activities. The key amino acids were marked by black triangles.

Supplementary Figure 9 CDS alignment of three isoforms (OsUAP2.1 and OsUAP2.2) from OsUAP2.

Supplementary Figure 10 Spatial structure comparison of isoforms from *OsUGP1* and *OsUSP*. **(A)** The structure comparison of *OsUGP1.1* (green) and *OsUGP1.2* (red). **(B)** The structure comparison of *OsUSP.1* (green) and *OsUSP.2* (red).

Supplementary Figure 11 *In vitro* and *in vivo* activities of *OsUAP2*. Enzymatic activities of two isoforms of *OsUAP2* based on ^1^H-NMR. **(A)** Forward activity: UTP + GlcNAc-1-P → UDP-GlcNAc + PPi. **(B)** Reverse activity: UDP-GlcNAc + PPi → GlcNAc-1-P + UTP. **(C)** Reverse activity: UDP-GalNAc + PPi → GalNAc-1-P + UTP. **(A–C)** Line 1, GST control. Line 2, protein of OsUAP2.1. Line 3, protein of OsUAP2.2. **(D)** The phenotype of wild type (WT), mutant (*osuap1*), and three independent complementary transgenic lines overexpressing *OsUAP2.1*. **(E)** The phenotype of wild type (WT), mutant (*osuap1*), and three independent complementary transgenic lines overexpressing *OsUAP2.2*.

Supplementary Table 1 Protein resource of UDPGP genes from 76 species.

Supplementary Table 2 Characteristics of the 454 UDPGPs in 75 species.

Supplementary Table 3 Orthogroups generated by McScanX.

Supplementary Table 4 Key amino acid residues affect the activity of UDPGP based on reported mutants.
