# Supplementary figures and images for: Global Analysis of UDP Glucose Pyrophosphorylase (UDPGP) Gene Family in Plants: Conserved Evolution Involved in Cell Death
Source: Front Plant Sci. 2021 Jun 10;12:681719. doi: 10.3389/fpls.2021.681719 (PMC8222925; doi:10.3389/fpls.2021.681719)

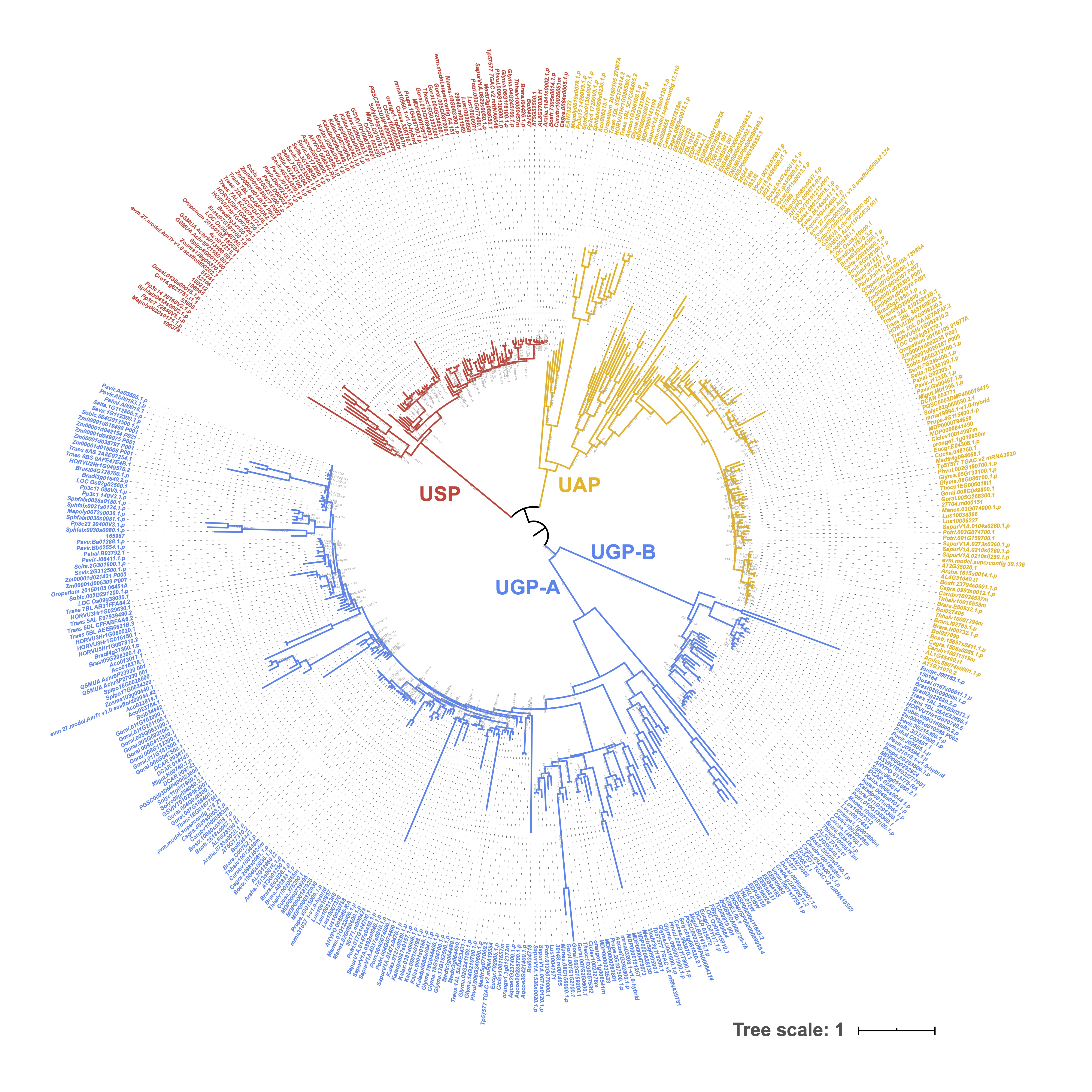

Supplement: Supplementary Figure 1 — Maximum likelihood tree of 75 species generated by IQ-Tree and branch supports using ultrafast bootstrap approximation/SH-aLRT/aBayes methods. UAP, UGP, and USP were marked in yellow, blue, and red, respectively. [file Image_1.JPEG]

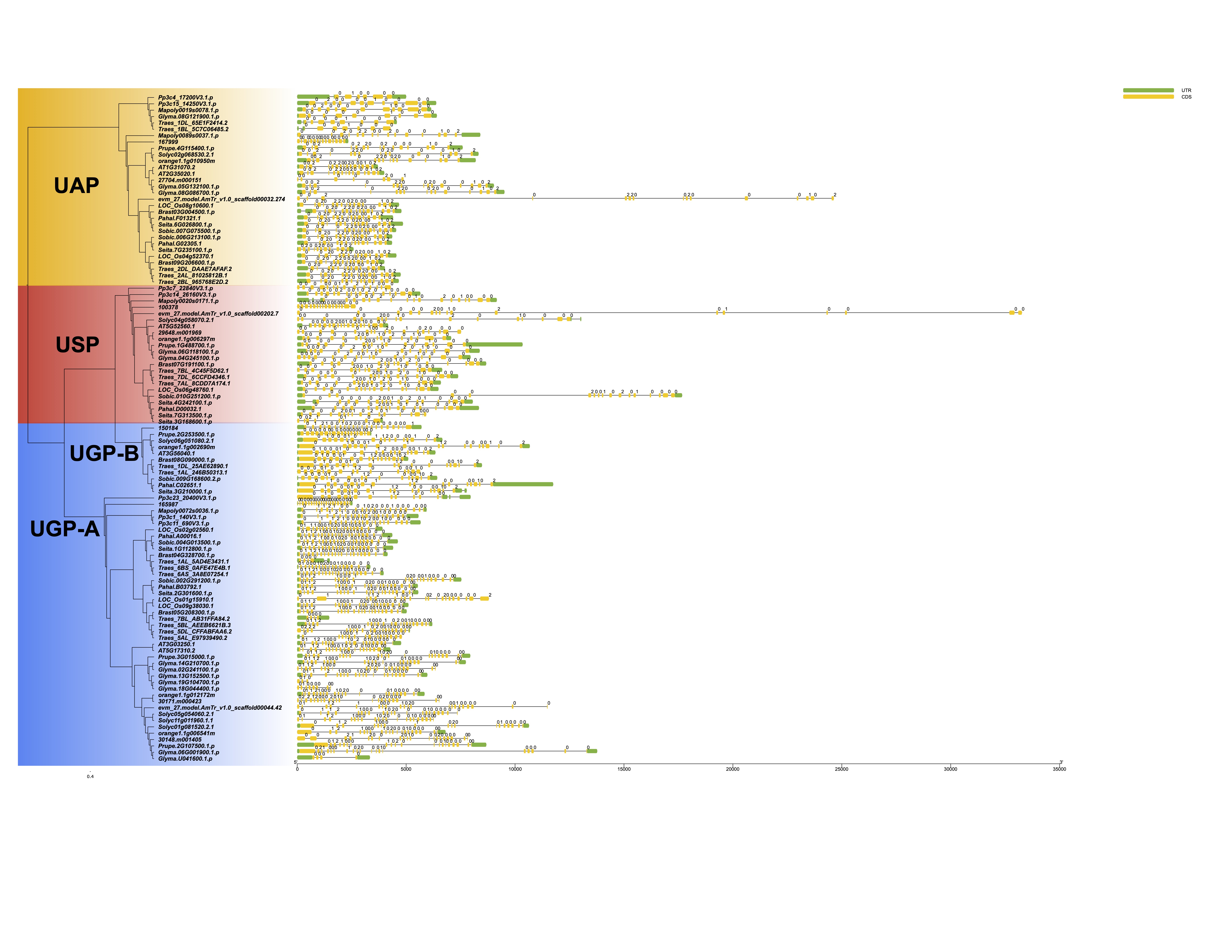

Supplement: Supplementary Figure 2 — Gene structures of 105 UDPGPs from 16 species. Phase 0 intron doesn’t interrupt a codon, phase 1 intron interrupts a codon between the 1st and 2nd bases, and phase 2 intron interrupts a codon between the 2nd and 3rd bases. [file Image_2.JPEG]

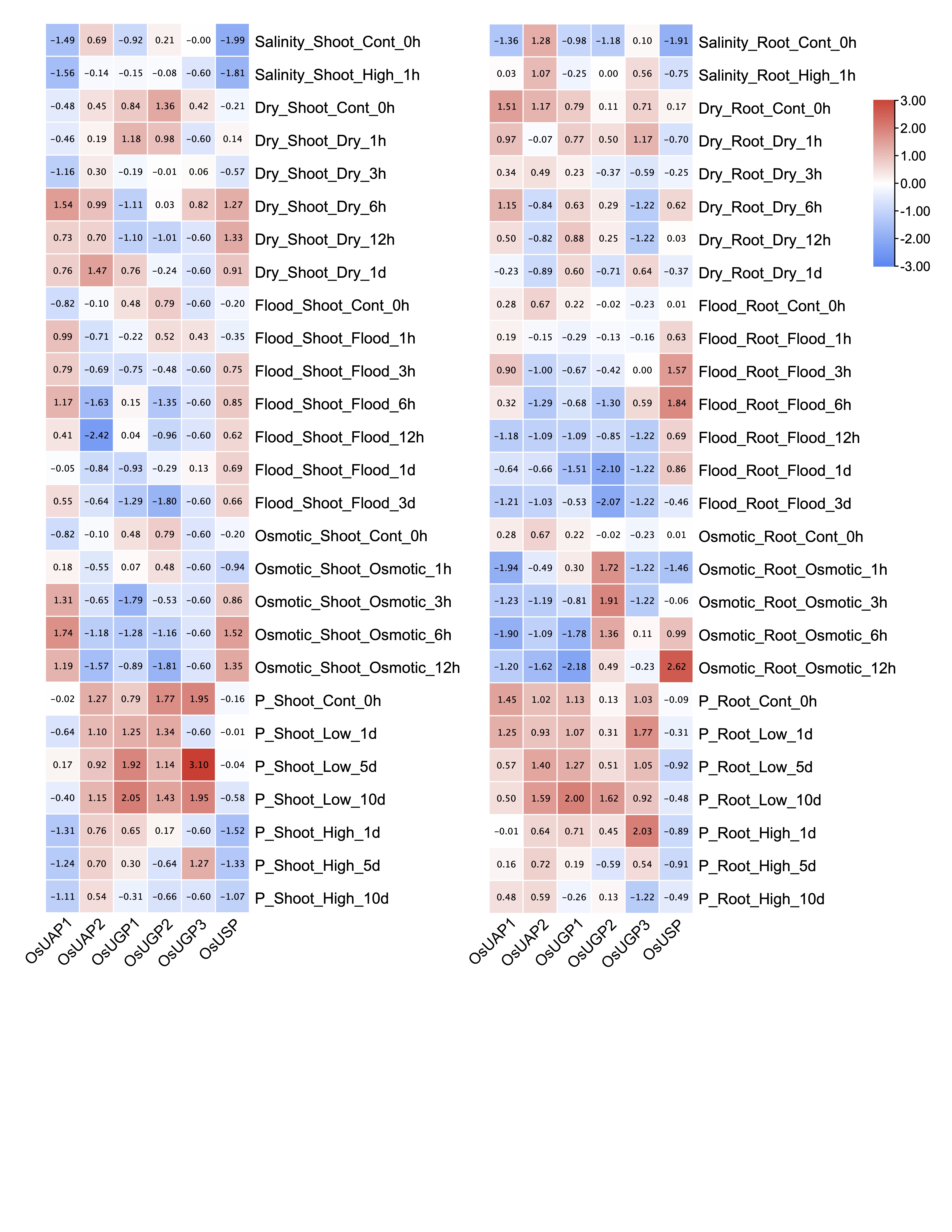

Supplement: Supplementary Figure 3 — Expression profile of UDPGPs of the shoot and root tissues under salinity (150 mM NaCl), dry (grown without medium), flood (completely submerged in medium), osmotic (0.6 M Mannitol), and P (3 mM KH2PO4) treatment from Oryza sativa. The scale on the right indicates the gene expression level transformed by log2(RPK). All the genes were normalized by columns. [file Image_3.JPEG]

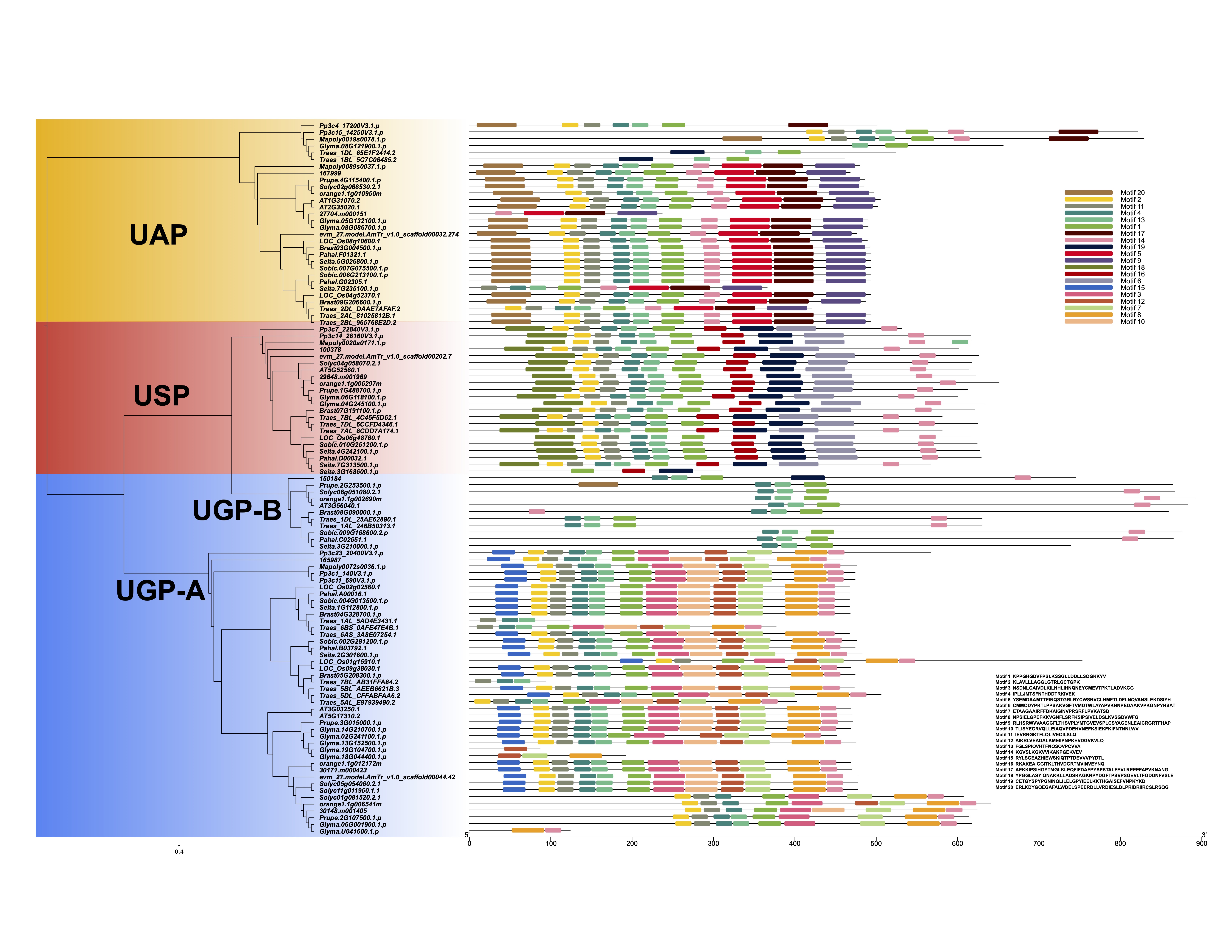

Supplement: Supplementary Figure 4 — Motif architectures of 105 UDPGPs from 16 species. [file Image_4.JPEG]

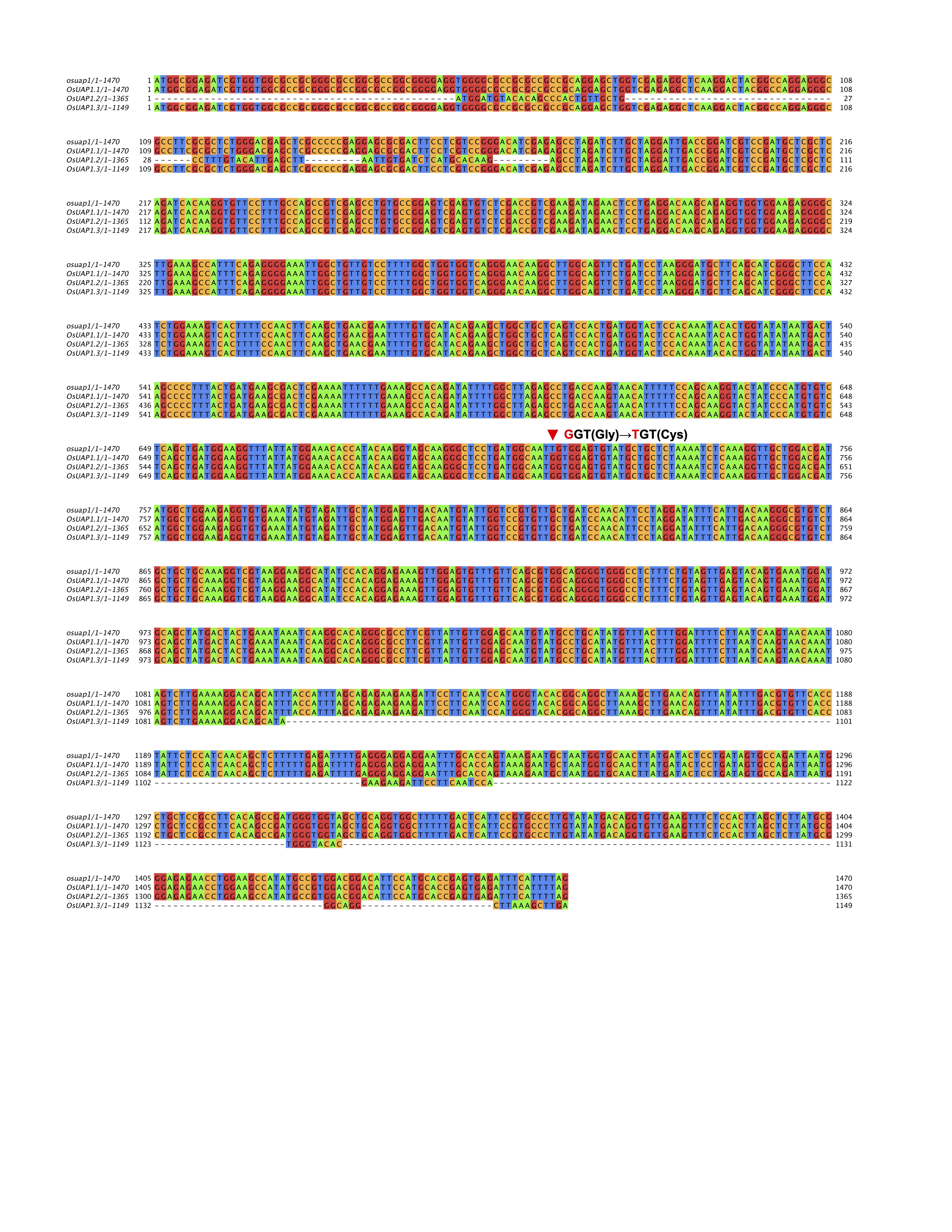

Supplement: Supplementary Figure 5 — CDS alignment of three isoforms (OsUAP1.1, OsUAP1.2, and OsUAP1.3) from OsUAP1. [file Image_5.JPEG]

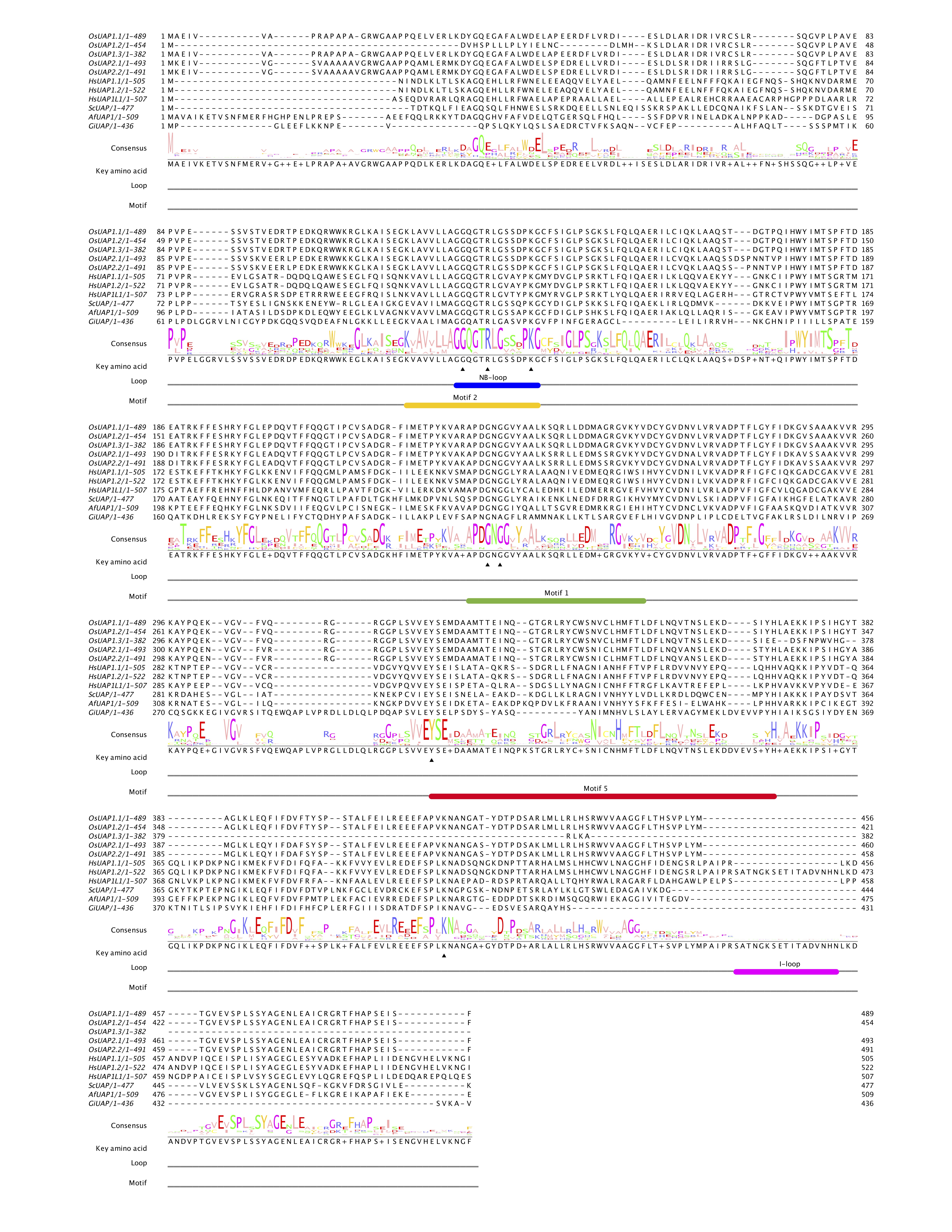

Supplement: Supplementary Figure 6 — Key amino acids affect UAP catalytic activities. The key amino acids were marked by black triangles. The black squares mean the deletion sites. [file Image_6.JPEG]

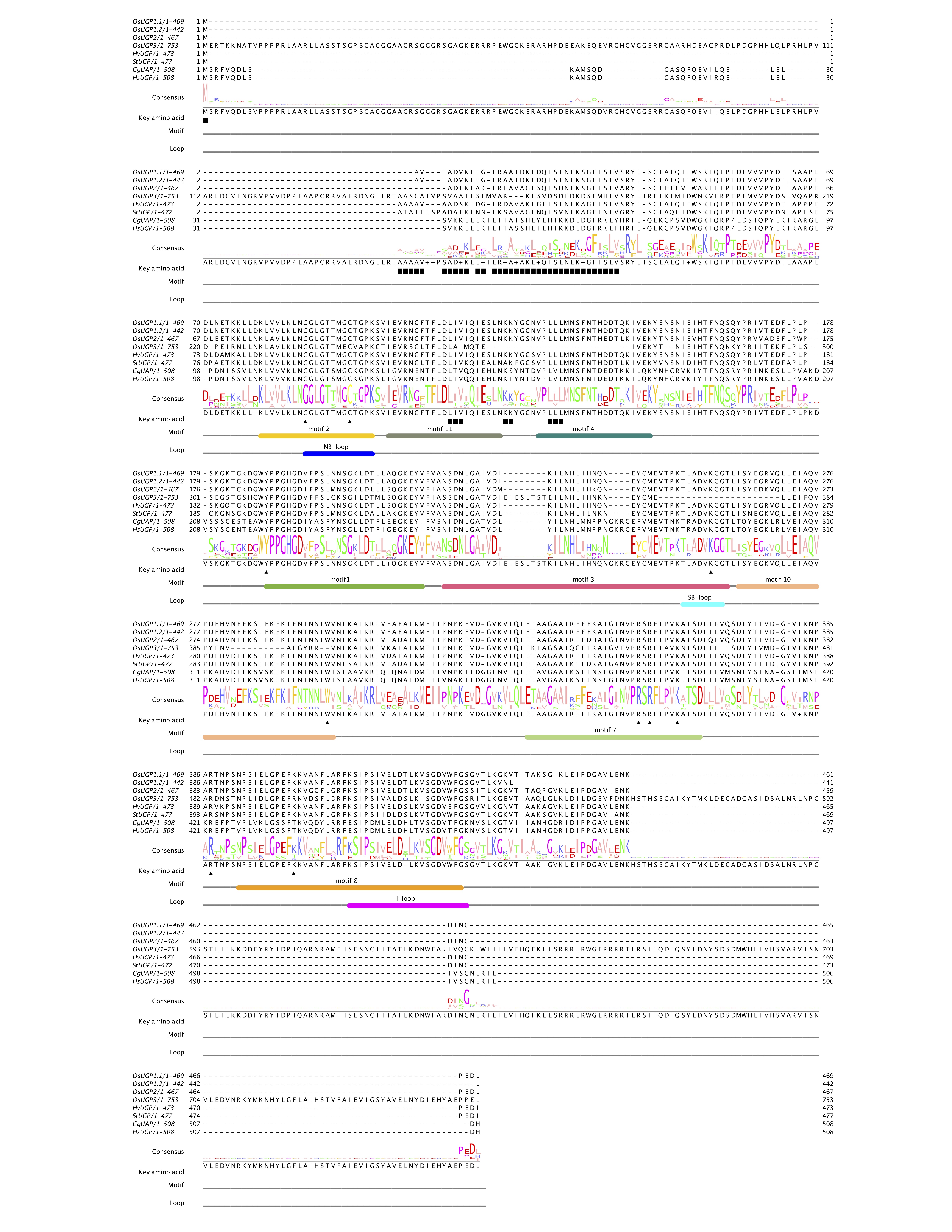

Supplement: Supplementary Figure 7 — Key amino acids affect UGP catalytic activities. The key amino acids were marked by dark triangles. [file Image_7.JPEG]

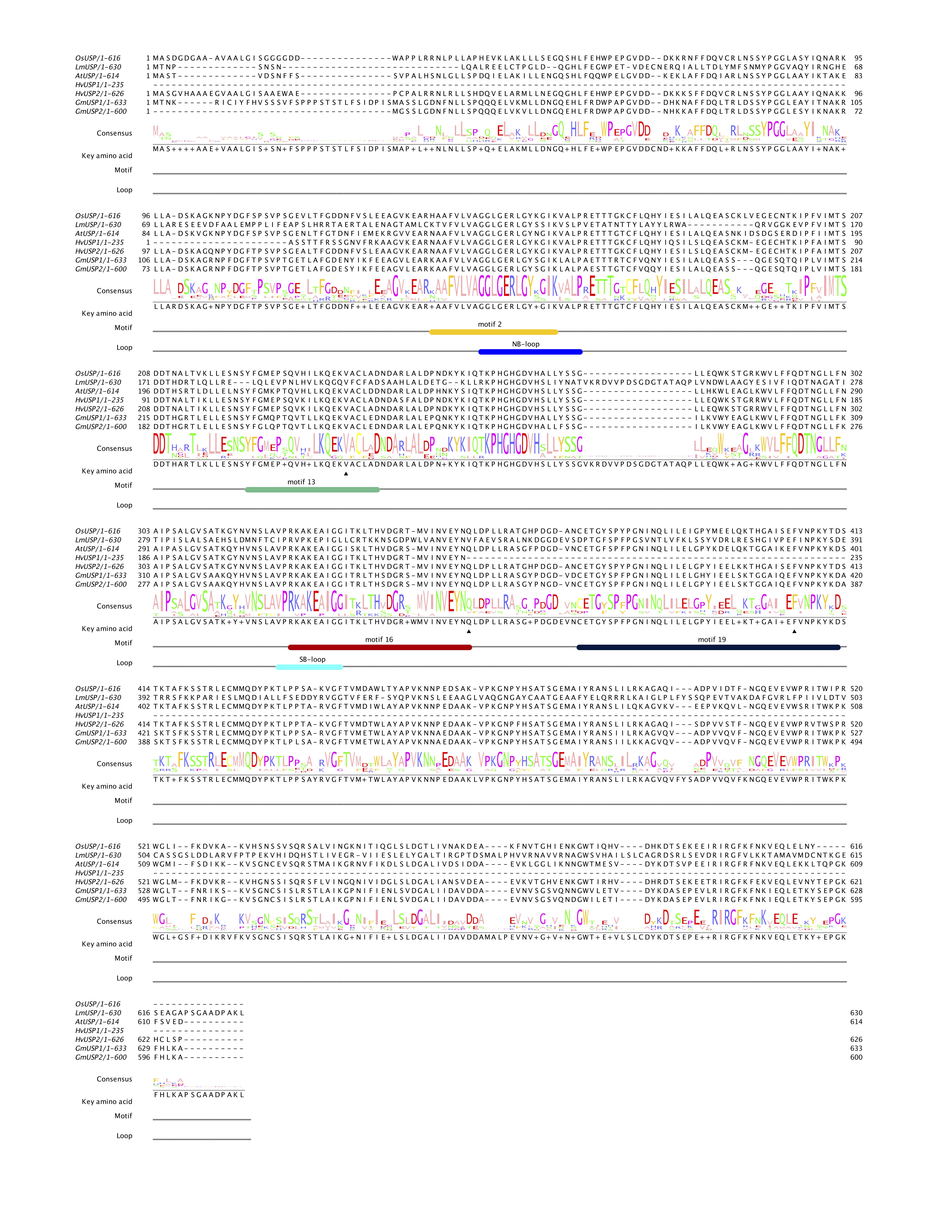

Supplement: Supplementary Figure 8 — Key amino acids affect USP catalytic activities. The key amino acids were marked by black triangles. [file Image_8.JPEG]

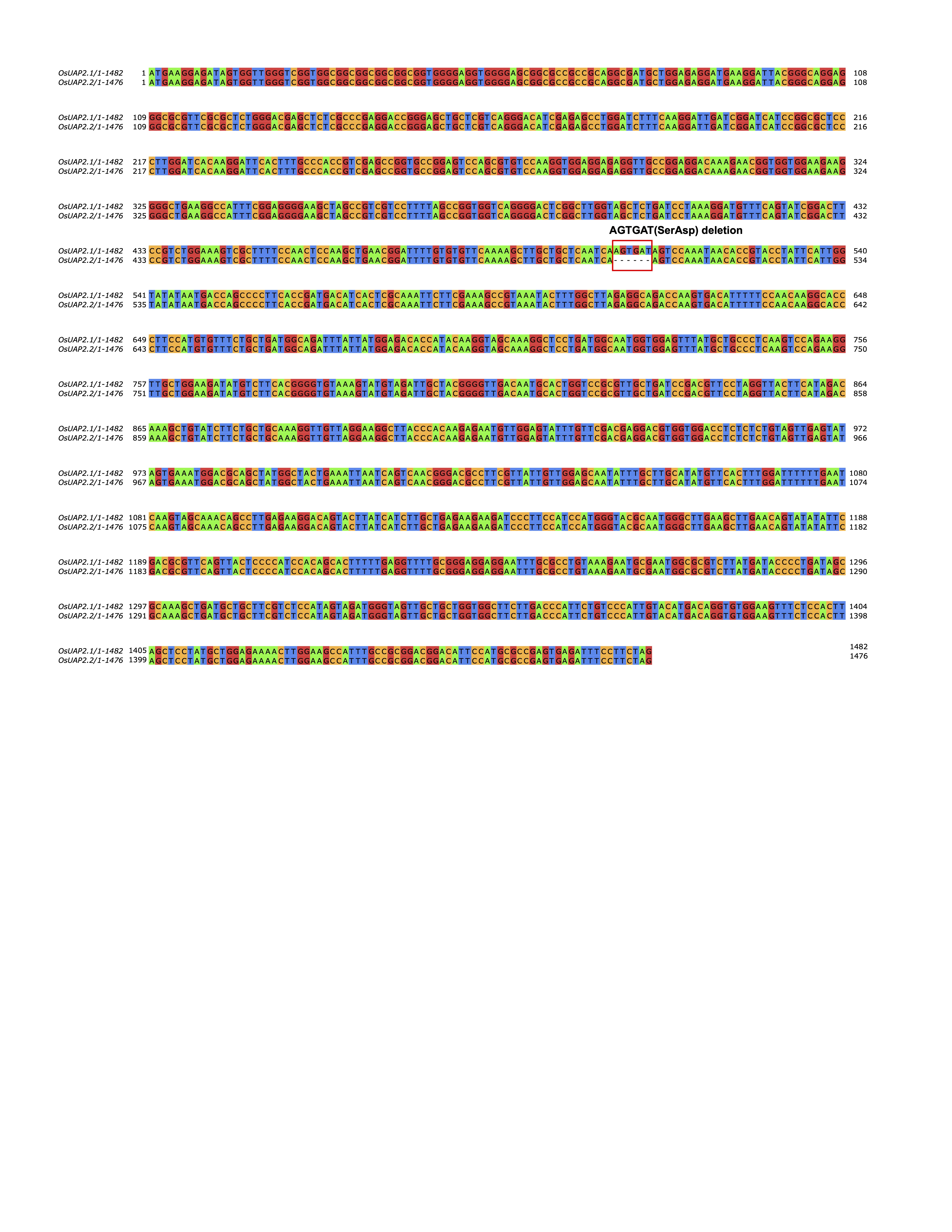

Supplement: Supplementary Figure 9 — CDS alignment of three isoforms (OsUAP2.1 and OsUAP2.2) from OsUAP2. [file Image_9.JPEG]

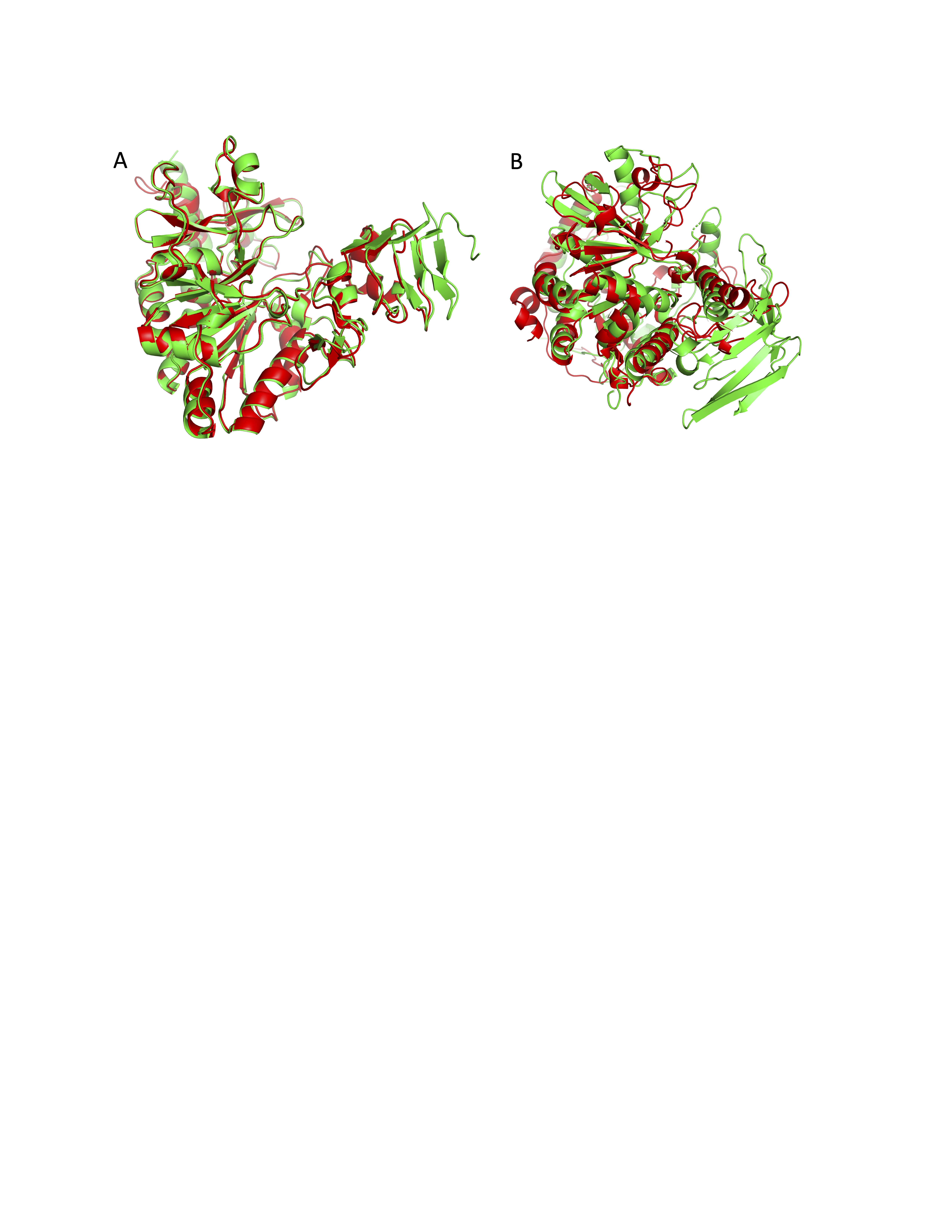

Supplement: Supplementary Figure 10 — Spatial structure comparison of isoforms from OsUGP1 and OsUSP. (A) The structure comparison of OsUGP1.1 (green) and OsUGP1.2 (red). (B) The structure comparison of OsUSP.1 (green) and OsUSP.2 (red). [file Image_10.JPEG]

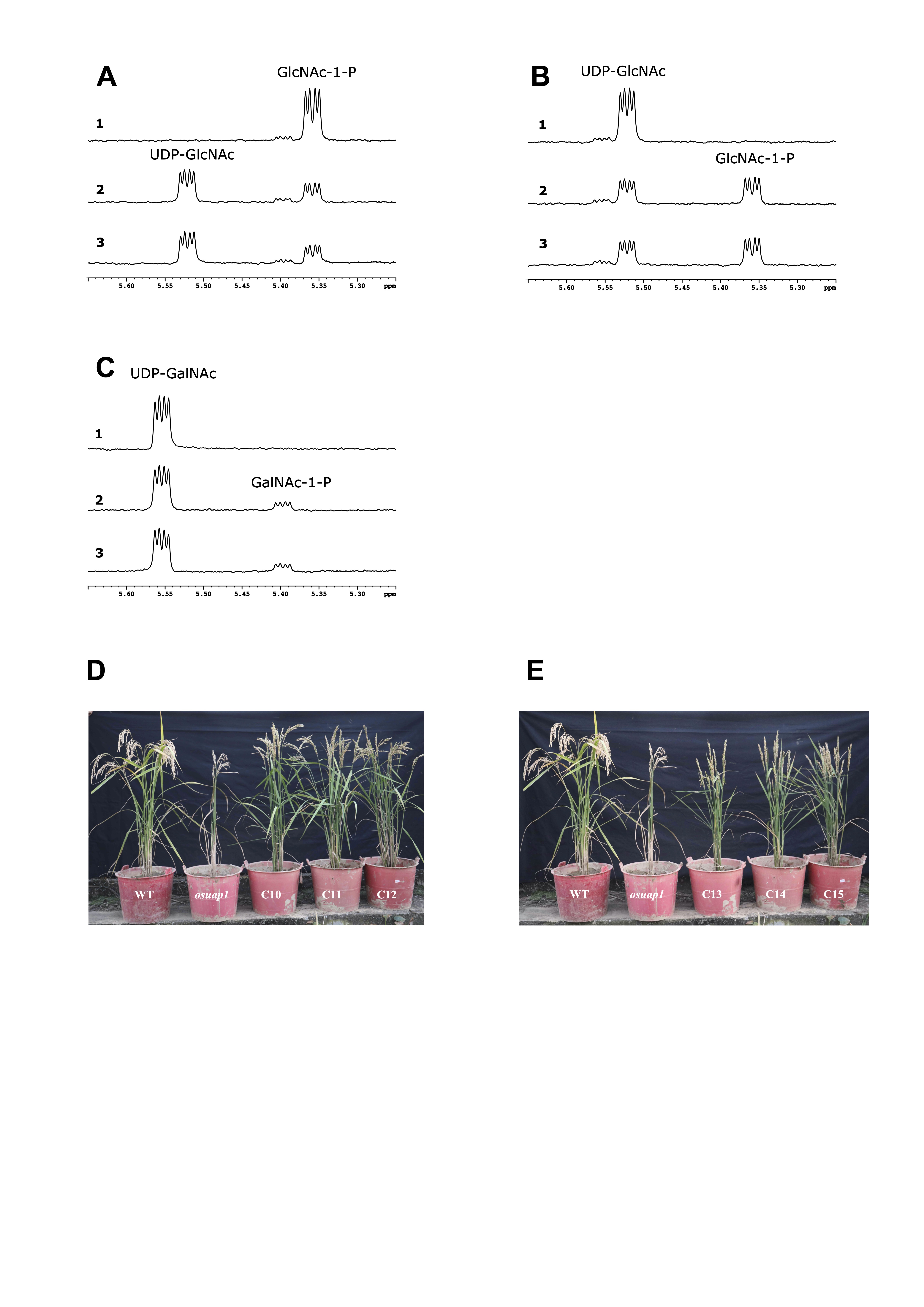

Supplement: Supplementary Figure 11 — In vitro and in vivo activities of OsUAP2. Enzymatic activities of two isoforms of OsUAP2 based on 1H-NMR. (A) Forward activity: UTP + GlcNAc-1-P → UDP-GlcNAc + PPi. (B) Reverse activity: UDP-GlcNAc + PPi → GlcNAc-1-P + UTP. (C) Reverse activity: UDP-GalNAc + PPi → GalNAc-1-P + UTP. (A–C) Line 1, GST control. Line 2, protein of OsUAP2.1. Line 3, protein of OsUAP2.2. (D) The phenotype of wild type (WT), mutant (osuap1), and three independent complementary transgenic lines overexpressing OsUAP2.1. (E) The phenotype of wild type (WT), mutant (osuap1), and three independent complementary transgenic lines overexpressing OsUAP2.2. [file Image_11.JPEG]
